# A let-7 microRNA-RALB axis links the immune properties of iPSC-derived megakaryocytes with platelet producibility

**DOI:** 10.1038/s41467-024-46605-0

**Published:** 2024-03-22

**Authors:** Si Jing Chen, Kazuya Hashimoto, Kosuke Fujio, Karin Hayashi, Sudip Kumar Paul, Akinori Yuzuriha, Wei-Yin Qiu, Emiri Nakamura, Maria Alejandra Kanashiro, Mio Kabata, Sou Nakamura, Naoshi Sugimoto, Atsushi Kaneda, Takuya Yamamoto, Hirohide Saito, Naoya Takayama, Koji Eto

**Affiliations:** 1 Department of Clinical Application, Center for iPS Cell Research and Application (CiRA), Kyoto University, Kyoto, Japan; 2 Department of Regenerative Medicine, Graduate School of Medicine, Chiba University, Chiba, Japan; 3 Department of Life Science Frontiers, Center for iPS Cell Research and Application (CiRA), Kyoto University, Kyoto, Japan; 4 Department of Molecular Oncology, Graduate School of Medicine, Chiba University, Chiba, Japan; 5 Institute for the Advanced Study of Human Biology (WPI-ASHBi), Kyoto University, Kyoto, Japan; 6 Medical-risk Avoidance Based on iPS Cells Team, RIKEN Center for Advanced Intelligence Project (AIP), Kyoto, Japan

**Keywords:** Pluripotent stem cells, Biotechnology, Platelets

## Abstract

We recently achieved the first-in-human transfusion of induced pluripotent stem cell-derived platelets (iPSC-PLTs) as an alternative to standard transfusions, which are dependent on donors and therefore variable in supply. However, heterogeneity characterized by thrombopoiesis-biased or immune-biased megakaryocytes (MKs) continues to pose a bottleneck against the standardization of iPSC-PLT manufacturing. To address this problem, here we employ microRNA (miRNA) switch biotechnology to distinguish subpopulations of imMKCLs, the MK cell lines producing iPSC-PLTs. Upon miRNA switch-based screening, we find imMKCLs with lower let-7 activity exhibit an immune-skewed transcriptional signature. Notably, the low activity of let-7a-5p results in the upregulation of RAS like proto-oncogene B (*RALB*) expression, which is crucial for the lineage determination of immune-biased imMKCL subpopulations and leads to the activation of interferon-dependent signaling. The dysregulation of immune properties/subpopulations, along with the secretion of inflammatory cytokines, contributes to a decline in the quality of the whole imMKCL population.

## Abstract (web summary)

The authors pioneered the iPSC-derived platelet transfusion in human. Here they employ miRNA switches, identifying RALB as a determinant of immune megakaryocytes and a marker for quality control, advancing standardization of iPSC-platelet production.

## Introduction

The first successful whole blood transfusion took place in 1818 by James Blundell^1^. This historic achievement was followed by Karl Landsteiner’s discovery of ABO blood types in 1901^2^ and the development of a platelet isolation device for transfusion by Emil J. Freireich in the 1950s-1960s^3^. Since then, donor-dependent platelet transfusion has become a standard healthcare practice despite persistent challenges of supply shortages resulting from various factors including viral contamination, alloimmune transfusion refractoriness, and the recent COVID-19 pandemic. In this context, we have adopted the strategy of ex vivo blood pharming using human induced pluripotent stem cells (iPSCs). We developed immortalized MK progenitor cell lines (imMKCLs) as the starting cell source for ex vivo iPSC-derived platelet (iPSC-PLT) manufacturing^4,5^. imMKCLs exhibit sustained proliferation over several months in the presence of doxycycline (DOX) and shed iPSC-PLTs after DOX removal, enabling the generation of over 10^11^ iPSC-PLTs in a turbulent flow-based bioreactor^6^. Building upon these achievements, we initiated the first-in-human clinical trial, iPLAT1^7–9^. While iPLAT1 demonstrated promising outcomes without significant adverse events, a transient increase in D-dimer levels and leukocyte count was observed after transfusion of the maximal dose^7^. These observations indicate the potential involvement of recently identified immune-skewed MKs^10,11^, raising concerns about the quality control of imMKCLs as master cells. Meanwhile, we noticed considerable variation in the quality of imMKCL clones with regards to their proliferation and iPSC-PLT production capacity^12^. Specifically, certain imMKCL clones displaying cellular senescence exhibited a diminished capacity to generate iPSC-PLTs, but the capacity could be restored through the knockdown of p53 and CDKN1A^12^. However, the molecular factors underlying this heterogeneity, which significantly hinder the efficiency and standardization of iPSC-PLT manufacturing, remain unknown.

MicroRNAs (miRNAs) are small non-coding RNAs that negatively regulate the stability and translation of the target mRNAs by binding with the complementary mRNA sequences. In hematopoietic cells, miRNAs have been identified as key players in cell fate decisions and functions^13^. Based on the evidence that DNA-based genetic circuits regulate protein expression depending on miRNA activities rather than miRNA expression^14^, we developed an innovative biotechnology method, miRNA switch, which enables the identification of specific cell types^15,16^. MiRNA switches can detect endogenous miRNA activity to distinguish heterogeneous cell populations without the need of antibody labeling. This technology has proven successful for various cell types, including iPSC-derived hepatocytes, endothelial cells, cardiomyocytes^15^, mouse embryonic stem cell (ESC)-derived neurons^17^, and undifferentiated human ESCs and iPSCs^18^. We therefore hypothesized that the miRNA switch can be employed to distinguish subpopulations among imMKCLs with heterogenous miRNA activities (i.e., a gradient variation in miRNA activity).

In this study, we screened a library containing 269 target miRNA switches, leading to the identification of let-7a-5p and let-7g-5p miRNAs with heterogenous activity among imMKCLs. By a transcriptional analysis of imMKCL subpopulations exhibiting either high or low let-7 activity (hereafter referred as let-7 high and let-7 low, respectively), we discovered that let-7 low imMKCLs exhibited an immune-skewed transcriptional signature. Further investigation unveiled the pivotal role of let-7a-5p and its downstream target, RAS like proto-oncogene B (*RALB*), in modulating the lineage determination of ‘immune’ MKs within imMKCLs. Importantly, we elucidated that the dysregulation of immune properties/subpopulations within imMKCLs, along with the secretion of inflammatory cytokines, results in a decline of their quality based on their arrested proliferation and deficient iPSC-PLT generation. This study provides valuable insights into the correlation between immune-biased MKs and their role in quality control during ex vivo iPSC-PLT manufacturing.

## Results

### Identification of miRNAs with endogenous activity in imMKCLs

**Fig. 1:**
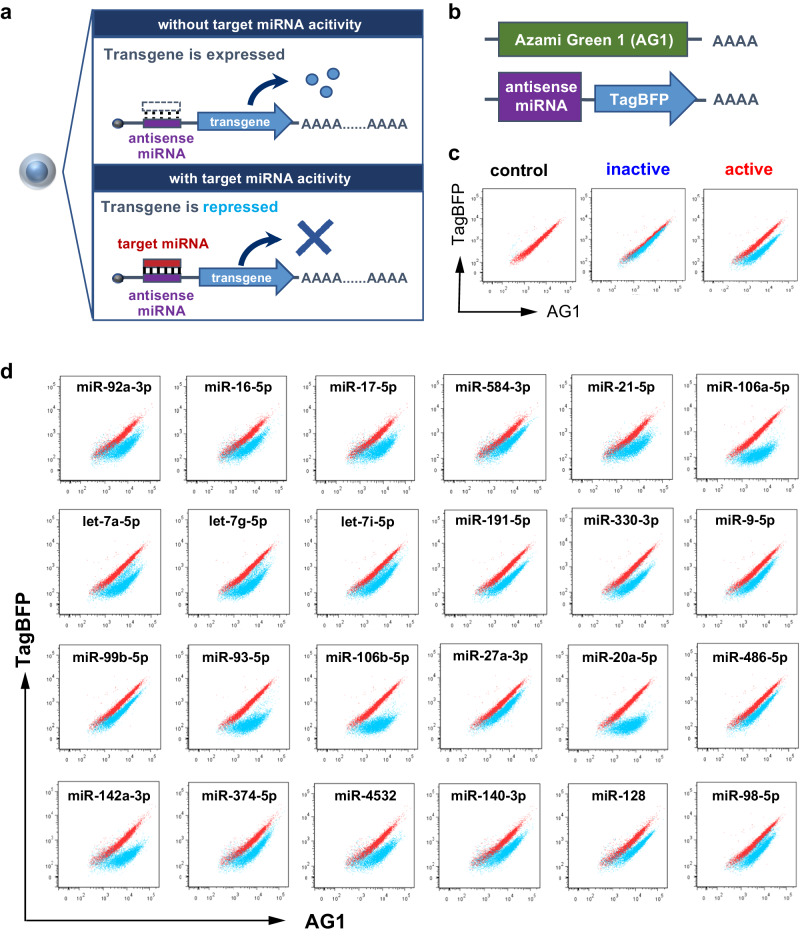
A miRNA switch-based screening identifies miRNAs with endogenous activity in imMKCLs. **a** A schematic illustration of the design of miRNA switches. The mRNA is composed of the antisense sequence of the target miRNA and a reporter transgene. In the presence of an active target miRNA, the expression of the reporter gene is repressed. **b** Design of miRNA switches for imMKCLs. A pair of mRNAs, one encoding TagBFP containing the antisense sequence of the miRNA target in the 5’ UTR and the other of Azami Green protein, were synthesized in vitro. The two mRNAs were co-transfected into imMKCLs via lipofection. 24 h after the transfection, the cells were analyzed by flow cytometry to identify the miRNA activity in imMKCLs through a screening. **c** Representative dot plots of target miRNA activity in imMKCLs. Active miRNA showed less TagBFP expression. **d** Flow cytometry analysis of the target miRNAs showing endogenous activity in imMKCLs. A miRNA switch-based screening was performed from a library containing 269 kinds of miRNA switches (Supplementary Table 1), and 24 kinds of miRNAs were identified with endogenous activity in imMKCLs.

MiRNA switch technology describes synthetic mRNA-based genetic circuits that regulate transgene expressions through miRNA activity^15,16^. We hypothesized that miRNA switches can identify imMKCL subsets with distinct miRNA activities, providing a valuable tool for exploring the heterogeneity within imMKCLs. Figure 1a depicts a schematic illustration of miRNA switch technology. We designed synthetic mRNAs that contain complementary sequences of target miRNAs in the 5′ untranslated region (UTR) and encode a reporter fluorescent protein to monitor endogenous miRNA activity. A pair of mRNAs were synthesized and co-transfected into imMKCLs, including Azami-Green 1 (AG1)-coding mRNA as a transfection control and TagBFP-coding mRNA containing the target miRNA sequence (Fig. 1b). TagBFP expression levels were measured by flow cytometry in cells with endogenous activity for a specific miRNA (Fig. 1c). A miRNA switch-based screening was conducted using a library containing 269 miRNA switches (Supplementary Table 1) and resulted in the identification of 24 types of miRNAs with endogenous activity in imMKCLs (Fig. 1d).

### Let-7 miRNA switches enrich immune-skewed imMKCLs

**Fig. 2:**
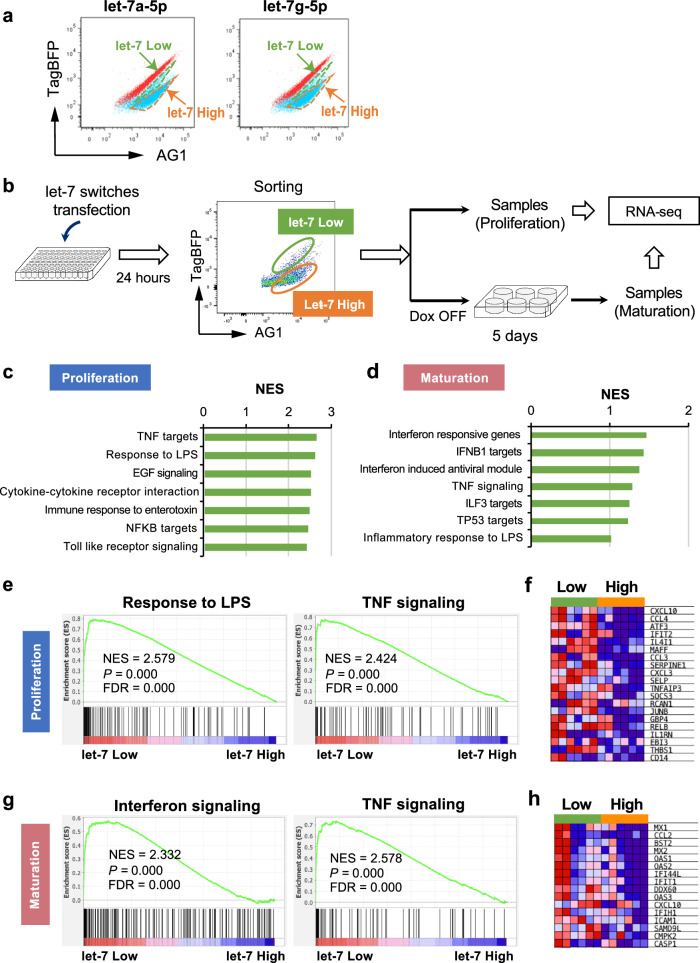
Let-7 miRNAs enable the identification of immune-skewed imMKCLs. **a** Heterogenous responsive activities in imMKCLs were identified with let-7a-5p or let-7g-5p miRNA switches, resulting in let-7 low-responsive and let-7 high-responsive subpopulations. **b** A schematic illustration of the bulk RNA-seq sampling workflow. Let-7a-5p and let-7g-5p switches were transfected into imMKCLs, followed by the sorting of let-7 low- and high-responsive cells by flow cytometry. Three distinct imMKCL clones (clone 7, clone 7–3, M35-1) were employed for the bulk RNA-seq analysis. Gene set enrichment analysis (GSEA) were performed to compare let-7 low (*n* = 6 independent biological samples) and let-7 high (*n* = 6) subpopulations. Bar graphs showing the GSEA results of the top enriched immune-related gene sets in let-7 low-responsive imMKCLs in the proliferation phase (**c**) and maturation phase (**d**). GSEA plots showing typical enriched immune-related gene sets in the proliferation phase (**e**) and maturation phase (**g**). Representative enrichment plots from each group are displayed with the normalized enrichment score (NES), the determined nominal (non-adjusted) *p*-value, and the false discovery rate (FDR) derived from GSEA software. Heatmaps of the top differentially expressed TNF targets of let-7 low- or high-responsive imMKCLs in the proliferation phase (**f**) and maturation phase (**h**). Source data are provided as a Source Data file.

Among the active miRNAs, we focused on let-7a-5p and let-7g-5p, as they showed heterogeneous activity among imMKCLs (Fig. 2a). We sorted subpopulations with high or low let-7 activity, with the let-7 low subset representing less than 5% of the total population. We confirmed that the let-7 activity levels in these populations correlated with their expression levels in imMKCL subpopulations (Supplementary Fig. 1a). To characterize molecular differences between the let-7 high and let-7 low subpopulations, we performed a bulk RNA-seq analysis using three different imMKCL clones (M35-1, clone 7, clone 7–3) in both the proliferation and maturation phases. The properties of clone 7 imMKCLs and the functionality of the derived iPSC-PLTs were reported previously^5,6^. Besides, clone 7 has been employed by multiple research groups for the investigation of megakaryopoiesis and thrombopoiesis^19–21^, highlighting its importance as a research tool. Clone 7–3, which is derived from clone 7 and displays an aged phenotype characterized by reduced proliferation and diminished iPSC-PLT production following repeated cultures, was also employed (Supplementary Fig. 1b). M35-1 was established from patient iPSCs for the iPLAT1 clinical trial due to its relatively superior expandability and iPSC-PLT productivity when compared with other competent patient-derived imMKCL clones^8^. Comparable iPSC-PLT generation was observed in the three clones for the let-7 low or let-7 high subpopulations, respectively (Supplementary Fig. 1c).

The sampling procedure for the bulk RNA-seq is illustrated in Fig. 2b. A differential gene expression analysis revealed many genes with altered expression profiles between let-7 high and let-7 low imMKCLs. To identify enriched transcriptional pathways in each subpopulation, we performed a gene set enrichment analysis (GSEA). Notably, we observed a series of immune-related gene sets that were significantly enriched in let-7 low imMKCLs in both the proliferation and maturation phases (Fig. 2c, d). Specifically, in the proliferation phase, gene sets in response to tumor necrosis factor (TNF), lipopolysaccharide (LPS), and enterotoxin were enriched in let-7 low cells, consistent with the reported properties of naïve mouse and human MKs with regard to sensing invading pathogens^22,23^. TNF-driven inflammation has also been suggested to induce platelet hyperreactivity in aging^24^. In the maturation phase, gene sets related to interferon signaling were significantly enriched in let-7 low cells. Some immune related properties were retained from the proliferation phase to maturation phase (Fig. 2e–h, Supplementary Table 2). These findings suggest that imMKCLs with lower let-7 activity preferentially enrich an immune-skewed MK phenotype. Our bulk RNA-seq analysis also identified the upregulation of several genes encoding chemokines and cytokines, including C-X-C motif chemokine ligand 10 (*CXCL10*), C-C motif chemokine ligand 2 (*CCL2*), and *CCL3*, in the immune-skewed let-7 low subpopulations of imMKCLs (Fig. 2f, h), indicating the consistency of multiple immune responses through the secretion of these molecules in human MKs^25^. Interestingly, elevated mRNA levels of these molecules have been reported in patients with severe acute respiratory syndrome (SARS) and middle east respiratory syndrome (MERS)^26^. Moreover, in COVID-19 patients, increased mRNA expression levels of *CXCL10*, *CCL2*, and *TNF* were correlated with an increased number of MKs^27,28^.

We further investigated whether immune MKs could be identified by let-7 miRNA switches in MK progenitors directly differentiated from human ESCs. We utilized CD34+ hematopoietic progenitor cells (HPCs) generated by our hPSC-sac method^29,30^ and performed a bulk RNA-seq analysis on two separate cell populations with different let-7a-5p and let-7g-5p activities (Supplementary Fig. 2a). A GSEA revealed similar enriched immune-related gene sets in let-7 low ESC-derived HPCs (Supplementary Fig. 2b, c, Supplementary Table 2), suggesting that the fate decision of immune-skewed MKs in MK development may occur as early as the hematopoietic progenitor stage.

### scRNA-seq reveals the heterogeneity and functional diversity of imMKCLs

**Fig. 3:**
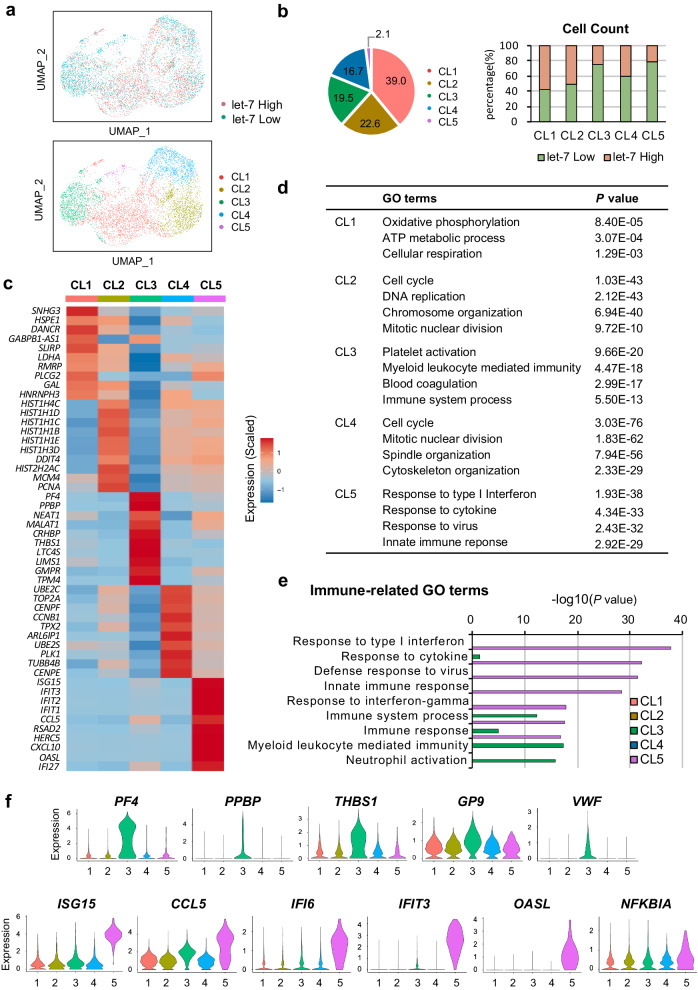
Single-cell RNA-seq reveals the presence of transcriptionally distinct immune-skewed subpopulations that are abundant in let-7a-5p low-responsive imMKCLs. **a** Visualization of imMKCLs (clone 7) by uniform manifold approximation and projection (UMAP) and colored according to let-7a-5p high/low-responsive imMKCLs and imMKCL sub-clusters. **b** The distribution of let-7a-5p low- and high-responsive cells in each cluster, and a bar plot showing the distribution of let-7a-5p low- and high-responsive cells in each cluster. **c** The relative expression level of the top ten DEGs in each cluster. **d** The representative terms of GO:BP enriched in each cluster. GO terms under the biological process category for upregulated differentially expressed genes (DEGs) in each cluster were analyzed by g:Profiler, *p*-values were determined by a default hypergeometric test and correction for multiple testing has been performed by the g:SCS algorithm. **e** Typical immune-related GO:BP terms in each cluster. **f** Violin plots showing the expression level of typical thrombopoiesis-related and immune-related genes in each cluster. Source data are provided as a Source Data file.

To further characterize the cellular heterogeneity of imMKCLs and explore the mechanism(s) underlying the enrichment of ‘immune’ imMKCLs by let-7 miRNA switches, we performed a single-cell (sc)RNA-seq analysis on let-7 low and high imMKCLs in the proliferation phase. Since both let-7a-5p and let-7g-5p miRNA switches demonstrated similar behaviors accordingly to the bulk RNA-seq analysis (Fig. 2), we focused our investigation on let-7a-5p. We identified five transcriptionally heterogeneous subpopulations of imMKCLs (Fig. 3a), with let-7 low imMKCLs being relatively concentrated in clusters 3 and 5 (Fig. 3b). We further determined the expression of the top differentially expressed genes (DEGs) and characterized the gene ontology (GO) terms enriched in each cluster (Fig. 3c, d).

Cluster 1 exhibited an enrichment of GO:BP (Biological Process) terms associated with oxidative phosphorylation, indicating their role in energy supply. Conversely, clusters 2 and 4 represented subsets of cycling MK progenitors with enriched GO:BP terms related to cell cycle and mitotic nuclear division. The identification of proliferating cell nuclear antigen (*PCNA*) and tropomyosin 4 (*TPM4*), two marker genes of cycling MKs, is consistent with recent studies on human bone marrow (BM) cycling MKs^31^. Cluster 3 highly expressed genes associated with “platelet activation” and “blood coagulation”, suggesting a subset of MK progenitors dedicated to platelet emergence. In particular, thrombopoiesis-associated genes, such as thrombospondin 1 (*THBS1*)^10^, von Willebrand factor (*VWF*)^32^, and platelet glycoprotein IX (*GP9*)^33^, were highly expressed in cluster 3 compared to the other clusters (Fig. 3f). Moreover, cluster 3 was enriched with genes related to the immune system process, suggesting that it may represent a subset of MK progenitors with functional duality. For instance, platelet factor 4 (*PF4*, also known as *CXCL4*), one of the top DEGs enriched in cluster 3, promotes blood coagulation (Fig. 3c)^34^ and is a crucial regulator in innate immunity through its activity on macrophages^35^ and neutrophils^36^. Additionally, pro-platelet basic protein (*PPBP*, also known as *CXCL7*), an activator of neutrophils against bacteria^37^, was also significantly upregulated in cluster 3.

Both clusters 3 and 5 may represent subpopulations of MK progenitors with highly upregulated potential immune-related genes. The annotated immune genes in clusters 3 and 5 are involved in immune system process and immune response according to the GO analysis (Fig. 3e). Cluster 5 highly expressed genes sets in response to type I interferon, cytokine, and virus, whereas cluster 3 appeared to be responsive to myeloid leukocyte-mediated immunity. CCL5, a key proinflammatory chemokine^38^, was one of the top DEGs enriched in cluster 5 (Fig. 3f). Notably, *CCL5* has been reported to promote proplatelet formation in thrombopoiesis^39^, and its expression was upregulated in cluster 3 compared to clusters 1, 2, or 4 (Fig. 3c, f). The interferon-stimulated gene for interferon-stimulated 15 (ISG15), a ubiquitin-like protein that can be covalently bound to host and viral proteins^40^, was identified as the top DEG in cluster 5. Given that the secretion of type 1 interferons from virus-infected cells is a hallmark of antiviral immunity^41^, our results indicated that cluster 5 represents the representative ‘immune’ MKs involved in antiviral functions. Recent in vivo models of naïve MKs have demonstrated the presence of ‘immune’ MKs, the hematopoietic stem cell (HSC) niche supporting MKs, and thrombopoiesis-biased MKs^10,11,31^. Notably, our results suggest that imMKCLs have a thrombopoiesis-biased subset (cluster 3) and a representative immune subset (cluster 5), similar to those found in endogenous human MKs.

### The inhibition of let-7a-5p activity drives the development of ‘immune’ imMKCLs

**Fig. 4:**
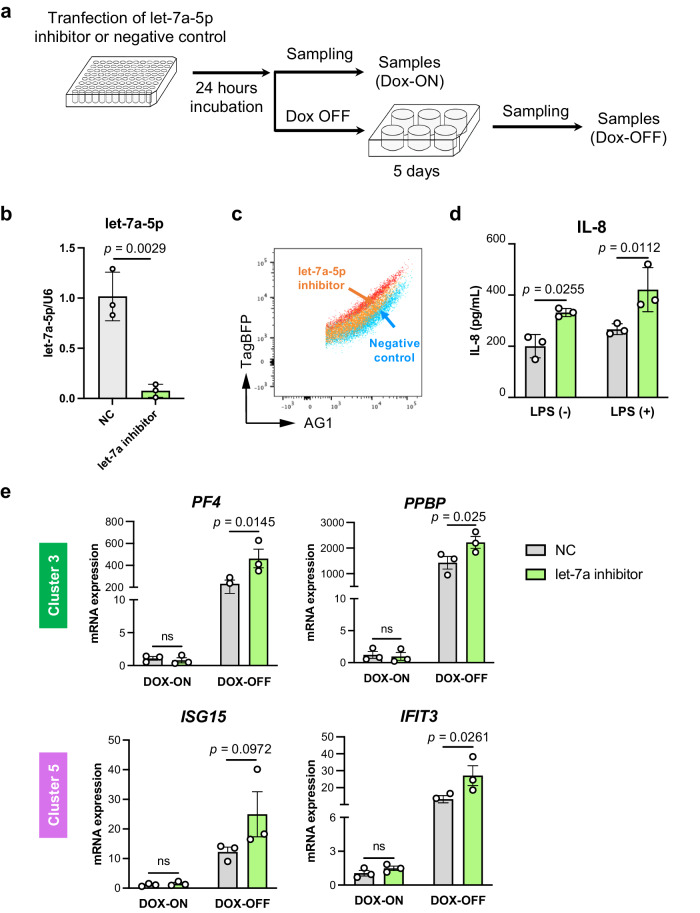
Let-7a-5p is a functional player in the development of immune-skewed subsets in imMKCLs. **a** A schematic illustration of the experimental workflow using a specific let-7a-5p inhibitor. **b** Let-7a-5p expression levels in imMKCLs (clone 7) treated with a negative control or let-7a-5p inhibitor. The expression levels were measured by qRT-PCR and normalized to an endogenous control, RNU6B (U6). **c** Let-7a-5p activity patterns were analyzed by FACS. **d** The inhibition of let-7a-5p in imMKCLs (clone 7) increased the mRNA expression levels of the marker genes identified in clusters 3 and 5 (Fig. 3). The mRNA expression levels were measured using qRT-PCR and normalized to GAPDH. **e** Let-7a-5p inhibition induced a greater secretion of IL-8 from imMKCLs at the proliferation stage (DOX-ON). After transfection of the let-7a-5p inhibitor or negative control, the imMKCLs were incubated for 24 h in the absence or presence of LPS (50 ng/mL) in the proliferation phase. Inflammatory molecules were measured by a cytometric bead array in the supernatant. Data are expressed as the mean ± SEM from three independent experiments. Unpaired two-tailed student’s *t*-tests were used to assess statistical significance. Source data are provided as a Source Data file.

We next investigated whether let-7 functionally drives the development of ‘immune’ subsets in imMKCLs by conducting loss-of-function experiments (Fig. 4a). We inhibited let-7a-5p expression/activity using a let-7a-5p inhibitor (Fig. 4b, c) and found enhanced gene expressions of several immune-related molecules identified in clusters 3 and 5, including *PF4*, *PPBP, ISG15*, and interferon induced protein with tetratricopeptide repeats 3 *(IFIT3)* (Fig. 4d). We also investigated whether the inhibition of let-7a-5p activity in imMKCLs alters the response to immune stimuli. The cells were stimulated with the pathogen receptor agonist LPS or control buffer. The supernatants were analyzed using a cytometric bead array kit by flow cytometry. We found that the inhibition of let-7a-5p in imMKCLs promoted the secretion of interleukin-8 (IL-8) (Fig. 4e), a critical proinflammatory chemokine^42^. IL-8 and its receptors have been shown to control MK proliferation and maturation^43^. These results are consistent with a previous report that found human cord blood-derived MKs produce IL-8 even in the absence of stimuli^44^. Collectively, we concluded that let-7a-5p is a functional player that modulates the development of ‘immune MKs’ among imMKCLs.

### RALB is a functional target of let-7a-5p in the development of immune-skewed imMKCLs

**Fig. 5:**
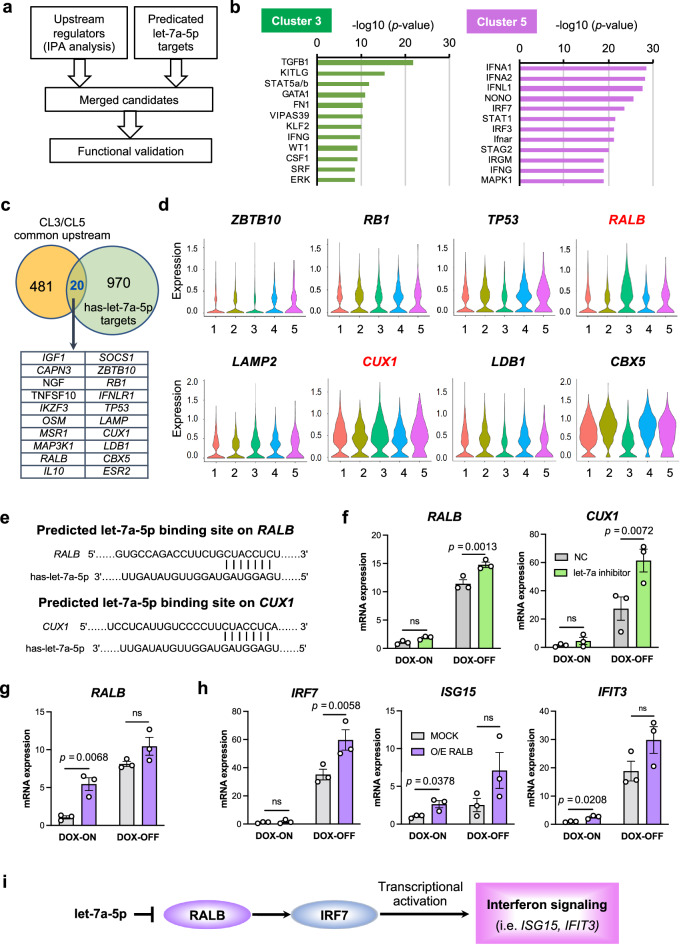
RALB is a target of let-7a-5p in the development of immune-skewed imMKCLs. **a** A flow chart of the upstream regulator analysis. **b** Bar plots showing the top 10 upstream regulators for cluster 3 and cluster 5 that were identified by the IPA based on scRNA-seq datasets. **c** The identification of eight potential upstream regulators by comparing the common upstream regulators of clusters 3 and 5 and predicted has-let-7a-5p targets. **d** Violin plots showing the expression levels of identified upstream regulators in each cluster. The regulators CUX1 and RALB showed increased expression levels in let-7a-5p low cells (clusters 3 and 5) and are colored red. The overlap *p*-values are calculated using Fisher’s Exact Test, and significance is generally attributed to *p-*values < 0.01. **e** The let-7a-5p binding site on *CUX1* and *RALB* predicted by TargetScan. **f** Let-7a-5p inhibition induced the mRNA expression of CUX1 and RALB in imMKCLs. **g** The lentiviral-mediated overexpression of RALB elevated the mRNA expression of RALB at both the proliferation (DOX-ON) and maturation stages (DOX-OFF). **h** The overexpression of RALB induced the expression of interferon signaling genes. **i** A schematic illustration showing how the let-7 miRNA-RALB axis modulates interferon signaling in imMKCLs. Data are expressed as the mean ± SEM from three independent experiments. Unpaired two-tailed student’s *t*-tests were used to assess statistical significance. Source data are provided as a Source Data file.

The preceding experiments revealed the critical role of let-7a-5p in immune-skewed imMKCLs. To further determine the underlying factors that contribute to immune-related outcomes, we conducted an ingenuity pathway analysis (IPA) to investigate the upstream regulators of cluster 3 and cluster 5 (let-7 low imMKCLs). Figure 5a shows a flowchart depicting the overall analysis design. IPA analysis identified the MK transcriptional factor *GATA1*^45^ and the myeloid development regulator *KLF2* as potential transcription activators in cluster 3 (Fig. 5b). Notably, KLF2 was shown to regulate host innate immune responses to polymicrobial infections^46^. On the other hand, known activators of virus-inducible cellular genes, such as interferon regulatory factor 7 (*IRF7*)^47^ and *IRF3*^48^, were identified as potential upstream regulators in cluster 5 (Fig. 5b). We also compared common upstream regulators of clusters 3 and 5 with the predicted let-7a-5p targets (Fig. 5c). Our analysis identified twenty candidate upstream regulators, of which eight candidates showed detectable expression levels in imMKCLs (Fig. 5d). Based on their increased expression levels in the enriched let-7 low clusters (clusters 3 and 5), we suspected that cut-like homeobox 1 (*CUX1*) and RAS like proto-oncogene B (*RALB*) are targets of let-7a-5p (Fig. 5e) and may contribute to the observed immune-related outcomes. Accordingly, the inhibition of let-7a-5p upregulated the mRNA expression levels of *CUX1* and *RALB* in both the proliferation and maturation phases (Fig. 5f). While the overexpression of *CUX1* did not cause significant effects (Supplementary Fig. 3), *RALB* overexpression upregulated the mRNA expression of *IRF7*, *ISG15*, and *IFIT3* (Fig. 5g, h, Supplementary Fig. 4a, b), but not the expression level of let-7a-5p (Supplementary Fig. 4c). On the other hand, despite being predicted as an upstream regulator of both clusters 3 and 5, *RALB* overexpression did not influence the expression of thrombopoiesis-related genes identified in Cluster 3 (Supplementary Fig. 4d), indicating that distinct let-7 targets may be responsible for regulating immune-related pathways and thrombopoietic pathways in imMKCLs. Combining our findings with the IPA (Fig. 5b), we propose that let-7a-5p tunes the immune properties of imMKCLs by targeting *RALB* (Fig. 5i).

### The dysregulated immune properties/subsets are associated with the quality of imMKCLs

**Fig. 6:**
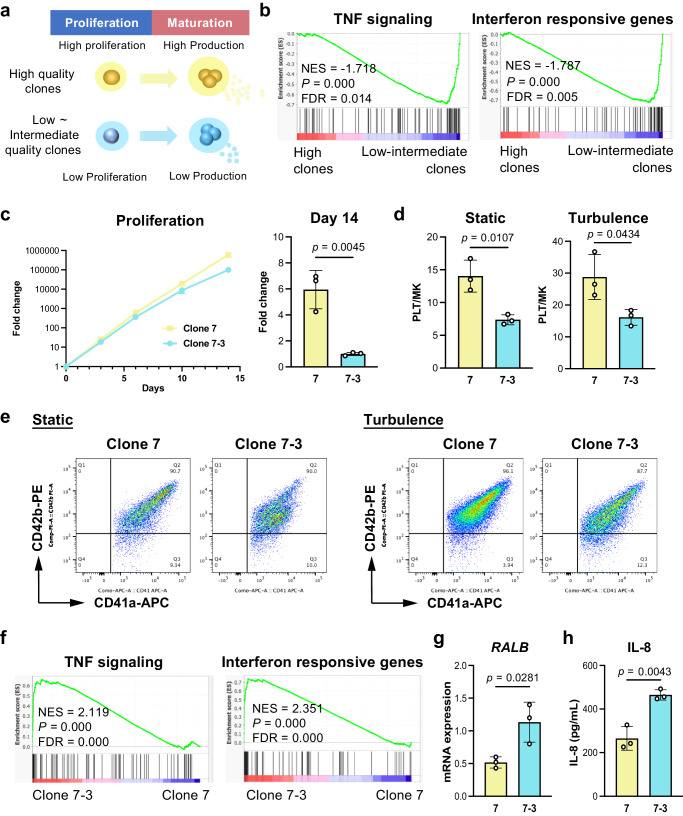
Dysregulated immune properties are associated with arrested proliferation and deficient iPSC-PLT generation of imMKCL clones. **a** A schematic illustration showing the correlation of proliferation and iPSC-PLT production by a previous study^12^. **b** GSEA were performed to compare high quality clones (*n* = 5) and Low ~intermediate clones (*n* = 7). GSEA plots showing enriched TNF signaling and interferon responsive gene sets in low-intermediate quality clones compared with high quality clones. **c** The proliferation and fold change of the cell count at day 14 of clone 7 and of clone 7–3. **d** iPSC-PLT production of clone 7 and of clone 7–3 in static or turbulent flow conditions. **e** Representative flow cytometry plots of iPSC-PLTs generated from clone 7 and clone 7–3 in static or turbulent flow conditions. **f** GSEA plots showing enriched TNF signaling and interferon responsive gene sets in clone 7–3. Representative enrichment plots from each group are displayed with the NES, non-adjusted *p*-value, and FDR derived from GSEA software. **g** Clone 7–3 shows elevated RALB mRNA expression compared with clone 7. **h** The secretion of IL-8 from clone 7 and clone 7–3 at the proliferation stage. Data are expressed as the mean ± SEM from three independent experiments. Unpaired two wo-tailed student’s *t*-tests were used to assess statistical significance. Source data are provided as a Source Data file.

We previously reported that imMKCL clones exhibiting slower proliferation rates at the proliferation stage are less capable of generating iPSC-PLTs (Fig. 6a)^12^. However, the factor(s) responsible for inducing the low quality of imMKCLs, along with the cellular senescent/aging transcriptional signature (Supplementary Fig. 5a, b), remains unclear. Therefore, we reexamined our GSEA of the different qualities of MKCLs utilized in our previous study^12^. Similar to the GSEA of the let-7 low and high populations (Fig. 2), we found TNF signaling and interferon responsive gene sets were significantly enriched in intermediate-to-low (low-intermediate) quality MKCLs (Fig. 6b, Supplementary Fig. 5c). We also found that clone 7 and clone 7–3, despite being derived from the same iPSC clone with the same genetic background^5,6^, display different characteristics with regards to proliferation and iPSC-PLT production in both static and turbulent flow conditions (Fig. 6c–e) and let-7a-5p activity patterns (Supplementary Fig. 6a). Clone 7–3 showed enriched TNF signaling and interferon responsive signaling compared to clone 7 (Fig. 6f, Supplementary Fig. 6b). These results suggest that the induced immune-related pathways are associated with low-quality imMKCLs. The GSEA further demonstrated that downregulated senescent tumor protein 53 (TP53) targets are enriched in clone 7 (Supplementary Fig. 6c), indicating the cellular senescence of clone 7–3. The mRNA expression of *RALB* and IL-8 secretion of clone 7–3 was higher compared with clone 7 (Fig. 6g, h). It is known that inflammatory cytokines are secreted by senescent cells with persistent DNA damage^49^. In addition to IL-8 secretion (Fig. 6h), genes encoding inflammatory cytokines and chemokines (*IFNB1*, *CXCL8*, *CXCL10*, *CXCL11*) also showed higher expression levels in clone 7–3 (aged clone) compared with clone 7 (younger clone) (Supplementary Fig. 6d). Notably, the addition of recombinant IL-8 into imMKCL culture resulted in diminished iPSC-PLT production (Supplementary Fig. 7a–c). Blocking IL-8 signaling with Reparixin^50^, a specific CXCR1/2 inhibitor, improved iPSC-PLT production (Supplementary Fig. 7d–g) without affecting proliferation. Furthermore, the administration of recombinant interferon-α2a led to lower proliferation rates (Supplementary Fig. 8a) as well as impaired iPSC-PLT production in a dose-dependent manner (Supplementary Fig. 8b, d). Interferon treatment elevated mRNA expression of the senescence marker *CDKN2A* as well as the interferon-responsive genes *ISG15* and *IFIT3* in imMKCLs (Supplementary Fig. 8c). Taken together, these finding suggest that the dysregulation of immune properties/subpopulation within imMKCLs, along with the secretion of inflammatory cytokines, leads to arrested proliferation and deficient platelet generation of the whole imMKCL population, potentially due to the upregulation of RALB levels. Moreover, these results emphasize the effect of immune cytokines on ex vivo iPSC-PLT manufacturing, thus bearing implications for clinical applications.

**Fig. 7:**
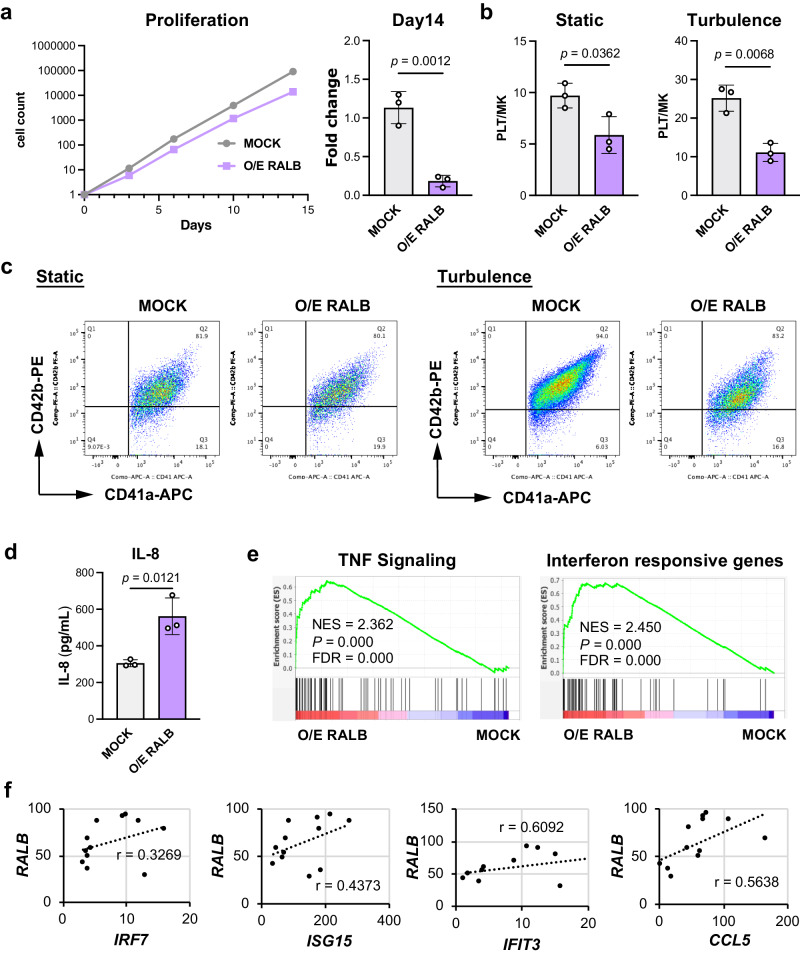
The overexpression of RALB diminishes the proliferation and iPSC-PLT generation capacity of imMKCLs, potentially by inducing the dysregulation of their immune properties. The lentiviral-mediated overexpression (O/E) of RALB leads to arrested proliferation (**a**) and deficient iPSC-PLT generation (**b**) in imMKCLs (Clone 7). **c** Representative flow cytometry plots of iPSC-PLTs generated from MOCK and O/E RALB in static or turbulent flow conditions. **d** IL-8 is secreted more by O/E RALB than by MOCK imMKCLs. **e** GSEA were performed to compare mock (*n* = 3) and RALB overexpressing cells (*n* = 3). GSEA plots showing enriched TNF signaling and interferon responsive gene sets in O/E RALB imMKCLs at the maturation stage. Representative enrichment plots from each group are displayed with the NES, non-adjusted *p*-value, and FDR derived from GSEA software. **f** A gene expression correlation analysis of RALB and immune-related genes. Data are expressed as the mean ± SEM from three independent experiments. Unpaired two-tailed student’s *t*-tests were used to assess statistical significance. Source data are provided as a Source Data file.

Intriguingly, our investigation of RALB revealed that the upregulation of *RALB* levels induced arrested proliferation, deficient iPSC-PLT production, and increased IL-8 secretion (Fig. 7a–d) as well as being accompanied by a cellular senescent/aging transcriptional signature (Supplementary Fig. 9a). We assumed that this mechanism could recapitulate the characteristics of low-quality imMKCLs. A bulk RNA-seq analysis revealed enriched TNF signaling and interferon response in *RALB-*overexpressing imMKCLs (Fig. 7e and Supplementary Fig. 9b), providing further support for our assumption. Additionally, RALB expression levels correlated with the expression levels of several interferon responsive genes in multiple imMKCL clones (Fig. 7f).

We employed two additional approaches to validate the effects of RALB. The small molecule inhibitor RBC8 (Supplementary Fig. 10a), which is described as a selective inhibitor against the GTPase Ral^51^, at 0.1 μM resulted in accelerated proliferation (Supplementary Fig. 10b) and ameliorated iPSC-PLT production (Supplementary Fig. 10c–e). siRNA-mediated *RALB* knockdown also enhanced iPSC-PLT production (Supplementary Fig. 10f, g). Although no significant difference was observed in IL-8 secretion (Supplementary Fig. 10h), RALB knockdown induced a decline in the mRNA expression levels of *IRF7*, *ISG15*, *IFIT3* (Supplementary Fig. 10i). Finally, the iPSC-PLTs generated from low quality clone (clone 7–3) showed decreased PAC-1 binding and *P*-selectin expression (Supplementary Fig. 11). These results collectively suggest that dysregulated immune properties/subsets within imMKCLs are associated with diminished imMKCL proliferation and iPSC-PLT generation, a phenotype potentially attributed to the upregulation of RALB expression.

Lastly, we verified our findings in an in vitro differentiated MK model derived from cord blood CD34^+^ cells (Supplementary Fig. 12a). The inhibition of let-7 had no significant effect on interferon signaling or platelet production (Supplementary Fig. 12b–d). However, the overexpression of *RALB* resulted in the upregulation of interferon-responsive genes and a reduction in the production of platelet-like particles (Supplementary Fig. 12e–g). While our results suggest imMKCL and primary MKs respond differently to let-7 inhibition, they indicate a common effect of RALB, highlighting the pivotal role of RALB in determining immune properties/subsets in MK development.

## Discussion

In this study, we applied miRNA switch technology to explore the heterogeneity of imMKCLs, a promising ex vivo source of iPSC-PLTs for transfusion therapy, at the proliferating stage. By screening a library containing 269 miRNA switches, we identified heterogenous let-7 miRNA activity among imMKCLs. By conducting bulk and scRNA-seq, we revealed that let-7 miRNA switches effectively enrich immune-skewed subpopulations. Our results demonstrate that the inability of let-7a-5p to suppress the expression of *RALB*, which lead to the activation of interferon-dependent signaling, is crucial for the lineage determination of immune-biased imMKCLs. While a senescence/aging signature is dominant in clone 7–3, we found the dysregulation of immune properties/subpopulations contributes to the secretion of inflammatory cytokines, resulting in a declined quality of the whole imMKCL population based on arrested proliferation and deficient iPSC-PLT generation. This senescence/aging related phenotype is potentially modulated by the let-7 microRNA/RALB/interferon signaling axis, as evidenced by RALB overexpression and knockdown. Consistently, we found the overexpression of RALB induced immune-skewed transcriptional phenotypes in a cord blood-derived MK model.

Non-coding RNAs, particularly miRNAs, play a crucial role in the post-transcriptional regulation of megakaryopoiesis^52^. For instance, miR-150^53^, miR-155^54^, and miR-146a^55^ have been proposed to act in MK lineage specification, while miR-125a-5p positively promotes proplatelet formation and platelet generation by targeting the expression of actin-bundling protein L-plastin^56^. Although the let-7 family is the most abundant miRNAs in megakaryocytic-erythroid progenitors^57^, its role in MK development has not been elucidated. Here we employed miRNA switch technology and observed the heterogeneous activities of let-7a-5p and let-7g-5p among imMKCLs. We discovered that immune-skewed phenotypes were preferentially enriched in imMKCLs with lower let-7 activity and characterized by the enhanced gene expression of immune-related molecules (i.e., *ISG15*, *IFIT3*; Fig. 3). The enriched gene sets in let-7 low imMKCLs (Fig. 2, Supplementary Table 2) partially overlapped with the enriched gene sets found in naïve lung MKs^22,58,59^. Interestingly, previous studies have suggested that the let-7 family is involved in the post-transcriptional control of innate immune responses to pathogenic agents^60^ and cytokine production by T lymphocytes in adaptive immunity^61^. In the present study, we observed that internal let-7 activity did not affect iPSC-PLT production (Supplementary Fig. 1c) and that the modulation of let-7 by mimics and inhibitors showed no discernible effect on iPSC-PLT production. These observations could be attributed to the intricate interplay of let-7 in regulating both the immune subset (cluster 5) and the thrombopoietic subset (cluster 3). We speculate that the inhibition of let-7 may compensate for any negative impact on iPSC-PLT production induced from the immune subset by influencing the thrombopoietic subset.

The classical view of megakaryopoiesis and thrombopoiesis has not provided insights about immune MKs in development. However, emerging evidence shows that lung-resident MKs in mice, particularly those in the non-vasculature area of the lungs, exhibit an immune-skewed transcriptional signature compared with BM-resident MKs^22,58^. Human embryonic MKs with immune characteristics were suggested to be generated along a distinct programmed trajectory^10^. Interestingly, our findings here show that heterogeneity is evident even within the immune-skewed MK population. We found imMKCLs contain ‘immune’ MKs in cluster 5, which displayed several immune programs (Fig. 3). The enriched gene sets in cluster 5 partially overlapped with those identified in human BM^31^. Cluster 3, on the other hand, exhibited functional duality in both thrombopoiesis and immunity. We suspect that cluster 3 contains two kinds of iPSC-PLT-generating MKs: one generating iPSC-PLTs for hemostasis specifically, and the other generating iPSC-PLTs with preferential immune functions. Cluster 3 may help explain why platelets have immune functions, while previously identified naïve immune MKs are mostly diploid and seemingly unable to produce platelets^11,22^.

Previous studies utilizing scRNA-seq have identified functionally distinct subsets of adult BM MKs^11,31^, including thrombopoiesis-biased MKs, immune MKs, and HSC niche-supporting MKs. However, the key molecular event(s) governing the development of the heterogenous ontology is poorly understood. The current study supports the functional diversity of imMKCLs, where lower let-7 activity enriches immune-skewed subpopulations. This heterogeneity may arise at or before the HPC stage (Supplementary Fig. 2). Furthermore, our findings reveal *RALB* as a functional target of let-7a-5p in modulating the lineage determination of ‘immune’ MKs within imMKCLs (Figs. 5, 7). *RALB* also drives the immune-skewed transcriptional phenotypes of cord blood-derived MKs (Supplementary Fig. 12), shedding light on the regulation of human immune MKs. RAL GTPases, encoded by *RALA* and *RALB*, are known for their roles in cell growth, granule secretion, and cancer metastasis^62^. Within the context of platelet biology, the activation of the RAL family in platelets occurs through α-thrombin-induced Ca^2+^/calmodulin binding to either RAL^63^. Additionally, the RAL family is recognized as critical regulators of *P*-selectin expression and platelet-leukocyte interactions in mice^64^. In cancer biology, evidence has highlighted the importance of the RALB/TBK1 pathway in cell autonomous survival by establishing a link between tumor formation and the innate immune response^65^. Considering the protective role of platelets in shielding cancer cells from immune surveillance by cytotoxic lympocytes^66^, it is reasonable to suspect that the RAL family serves as the molecular conduit connecting MKs/platelets and cancer. While another identified upstream regulator candidate, *CUX1* (Fig. 5), is essential for modulating endothelial senescence^67^, our study highlighted the involvement of *RALB* in the senescence process during hematopoiesis. For ex vivo iPSC-PLT manufacturing, our findings indicate *RALB* may serve as a hallmark/predictor of imMKCL quality. Indeed, some low-quality imMKCL clones exhibited elevated *RALB* expression levels (Fig. 6g, Supplementary Fig. 5d). In addition, a decline in imMKCL quality, along with senescent phenotypes, was observed following the overexpression of RALB (Fig. 7).

The success of our ex vivo manufacturing strategy for iPSC-PLTs predominantly relies on maintaining high-quality imMKCLs preserved in a master cell bank (MCB)^8,9^. To meet the demand for platelet transfusion therapy, which typically requires 2–3 × 10^11^ platelets per standard dose^68^, it is mandatory to ensure a substantial quantity of imMKCLs. However, the heterogeneity of imMKCLs poses a considerable challenge to achieving efficient and standardized iPSC-PLT manufacturing and may result in the heterogenous functionality of iPSC-PLTs^69^. In the iPLAT1 study, which used patient-derived imMKCLs (originally an iPSC clone derived from T cells of the patient), a slightly elevated white blood cell count and D-dimer level, which were observed after the transfusion of 10^11^ iPSC-PLTs in cohort 3^7^, indicated the possibility of immuno-thrombosis and the existence of immune subpopulations within imMKCLs. In the present study, we discovered a significant correlation between the immune properties/subpopulations of imMKCLs and their capacity to generate iPSC-PLTs. Whilst the immune subpopulations of MKs have been indicated as a genetically programmed ontogeny^10^, our findings suggest that the dysregulation of immune subpopulations/properties can have a significant impact on the overall imMKCL population, resulting in a decline of imMKCL quality. This decline is potentially mediated through the let-7 miRNA/RALB/interferon signaling axis, as evidenced by the immune-skewed transcriptional signatures and deficient iPSC-PLT production upon *RALB* overexpression (Figs. 5–7). Furthermore, our study revealed that utilization of the small molecule inhibitors RBC8 (Supplementary Fig. 10) and Reparxin (Supplementary Fig. 7) against RALB and IL-8 signaling favors iPSC-PLT production. The consistent efficacy of these small molecules across various clones suggests their universal functionality, highlighting their potential applicability in future clinical settings. Taken together, this study highlights the significance of considering the immune/senescent attributes of donor cells and allogenic imMKCL MCB in future iPSC-PLT transfusion therapies, as they profoundly impact both the quantity and quality of the produced iPSC-PLTs. Thus, this study provides important insights into the standardization of iPSC-PLT generation towards industrial-scaled manufacturing.

## Methods

### Cells

The human iPSC lines TkDN-Sev2 and T-1 were established in house^70^. imMKCLs (clone 7, clone 7–3, M35-1) were induced by DOX-inducible defined factors from human iPSCs and employed in previous studies^5,6^. The human ESC line KhES-3 was obtained from the Institute for Frontier Medical Sciences, Kyoto University (Kyoto, Japan). The cord blood-derived CD34+ cells were provided by the Japanese Red Cross Society Kanto-Koshinetsu Blook Blood Center. The use of all cells was approved by the ethics committees at Kyoto University and Chiba University.

### Cell culture

The imMKCLs (clone 7, clone 7–3, M35-1) were cultured as described before^6^. Doxycycline was used to control the proliferation and differentiation stages.

### MicroRNA switches

miRNA-responsive mRNAs (miRNA switches) were generated using a MegaScript T7 kit (Ambion) as described previously^15^. The miRNA switches were encoded on modified mRNA to post-transcriptionally regulate TagBFP, a blue fluorescent protein, in response to the activity of an arbitrary miRNA (miR-X) expressed in imMKCLs. TagBFP-coding mRNA includes the target miR-X sequence in 5′ UTR such that the expression level of TagBFP is suppressed in response to increased miR-X activity. Azami Green 1 (hmAG1)-coding mRNA served as the transfection control (Fig. 1B).

### miRNA switch-based screening

The miRNA switch-based screening was performed using an original constructed library (total of 269 miRNA switches). Two different mRNA coding for TagBFP and AG1, respectively, were co-transfected into the cells. The transfection of miRNA switches was performed as previously described^15^. Briefly, in the proliferation phase of imMKCLs, the reverse transfection of 150 ng mRNAs (75 ng each) was done with StemFect mRNA (Stemgent) in 50 μL of the cell suspension (1 × 10^6^ cells/mL) for 30 min, followed by dilution to 200 μL using culture medium in 96-well plates. After 24 h, flow cytometry was performed using a LSRFortessa (BD Biosciences, San Jose, CA, USA). Candidate miRNA switches were selected based on the active responsive pattern of imMKCLs to the target miR-X (“active” in Fig. 1c). MiRNAs that did not show strong responsive activities in imMKCLs were defined as “inactive” (Fig. 1c). For let-7a-5p and let-7g-5p, we individually transfected the candidate switches with control hmAG1-coding mRNA into imMKCLs. Following a 24 h incubation, the cells were sorted using a BD FACSAria II and subjected to further investigation.

### Cell sorting and flow cytometry

Cells were suspended in staining medium, incubated for 30 min with appropriate antibodies on ice in the dark, and sorted or analyzed using a BD FACSAria II. Platelet count was determined as previously described^6^. The following antibodies were used for the flow cytometry: allophycocyanin (APC)-conjugated anti-CD41a (integrin aIIbb3 complex: HIP8 clone) (Biolegend, San Diego, CA), phycoerythrin (PE)-conjugated anti-CD42b (GPIbα) (eBioscience, San Diego, CA), and PE-conjugated anti-CD41a (HIP8 clone) (Biolegend). The gating strategies are provided in the Supplementary Fig. 13.

### Reverse transcription and real-time PCR

Total RNA was extracted using the microRNeasy Micro Kit or microRNeasy Mini Kit (Qiagen, Hilden, Germany) and reverse-transcribed using SuperScript VILO™ Master Mix (Thermo Fisher Scientific). qPCR was carried out with SYBR™ Green PCR Master Mix (Applied Biosystems, Foster City, CA, USA) using a StepOnePlus system (Thermo Fisher Scientific). *GAPDH* was used as the internal control. The primer sets used are listed in Supplementary Table 3. Let-7a-5p and let-7g-5p miRNA reverse transcription was performed using a TaqMan MicroRNA Reverse Transcription Kit (Thermo Fisher Scientific) and miRNA specific stem-loop RT primers according to the manufacturer’s instructions. qPCR was carried out with TaqMan Fast Advanced Master Mix (Thermo Fisher Scientific) using the StepOnePlus system. The let-7 expression levels were determined relative to RNU6B using specific TaqMan probes.

### Analysis of cytokine secretion by imMKCLs

Cytokine secretion in culture supernatants was measured using the human inflammatory cytokine cytometric bead array kit (BD Biosciences) according to the manufacturer’s guidelines. Briefly, bead populations with distinct fluorescence intensities coated with capture antibody proteins were mixed with PE-conjugated detection antibodies and recombinant standards or test samples, and then incubated to form sandwich complexes. After acquiring sample data by flow cytometry, the cytokine concentrations were calculated using FCAP Array^TM^ software (BD Biosciences).

### Transfection of let-7a-5p inhibitor

An mirVana let-7a-5p-specific inhibitor and negative control were purchased from Thermo Fisher Scientific and used according to the manufacturer’s instructions. The transfection procedure was performed using a Stemfect RNA Transfection Kit (ReproCell, Yokohama, Japan).

### Lentiviral production

The use of viral vectors was approved by committees at Kyoto University and Chiba University. The full-length coding sequences of human CUX1 and RALB were cloned into the lentiviral vector CS2-Ubic-IG-GFP or CS2-Ubic-IB. Lentiviral production using the 293 T system was described previously^4^.

### Bulk RNA-sequencing analysis

Total RNA was extracted using the microRNeasy Micro Kit. RNA-seq libraries were prepared according to the manufacturer’s protocol. Briefly, ~10 ng of total RNA was used for the cDNA synthesis using a SMART-Seq v4 Ultra Low Input RNA Kit for sequencing (Takara Bio). cDNA was fragmented using an S220 Focused-ultrasonicator (Covaris, Woburn, MA, USA). The cDNA library was then generated using a NEBNext® Ultra™ DNA Library Prep Kit for Illumina (New England BioLabs, Beverly, MA, USA). Finally, the NEBnext library size was estimated using a bioanalyzer with an Agilent High Sensitivity DNA Kit. Sequencing was performed using a HiSeq2500 (Illumina) or a NextSeq 500 (Illumina) platform with a single-read sequencing length of 60 bp. TopHat (version 2.1.1) was used to map to the reference genome (UCSC/hg19) with annotation data from iGenomes (Illumina). Gene expression levels were quantified using Cuffdiff (Cufflinks version 2.2.1) and expressed as fragments per kilobase of exon per million mapped sequence reads (FPKM).

### Gene sets enrichment analysis (GSEA)

A GSEA was applied to screen pathways enriched in (1) let-7 (let-7a-5p and let-7g-5p) low or high imMKCLs (M35-1, clone 7, clone 7–3), (2) let-7 low imMKCLs of clone 7–3 or of clone 7, and (3) MOCK or RALB-overexpressing imMKCLs (clone 7) based on bulk RNA-seq datasets. The plots were generated using GSEA software (version 4.2.3) from the Broad Institute. We also re-analyzed bulk RNA-seq datasets from high-, intermediate- and low-quality MKCL clones^12^. The normalized gene expression matrix was input for the GSEA, and FDR and *P*-values < 0.05 were considered statistically significant.

### Chromium 10x single-cell RNA-seq library construction

imMKCLs (clone 7) with distinct let-7a-5p activity were sorted separately and resuspended at 1000 cells/μL in PBS with 0.4% BSA (Fig. 3). The cells were loaded onto a Chromium Next Gel Beads-in-EMulsion (GEMs) Chip G Single Cell Kit (10 x Genomics, USA). GEM generation and barcoding, reverse transcription, and cDNA generation and library construction were performed following the manufacturer’s protocol (Chromium Next GEM Single Cell 3’ Reagent Kits v3.1 Dual Index, 10 x Genomics). Dual-indexed, single-cell libraries were pooled and sequenced in paired-end reads on a Novaseq6000 (Illumina).

### Bioinformatics analysis

10 x Genomics-derived datasets were collected, and quality control was performed to filter out low-quality and contaminated cells. Generally, reads were pre-processed with the Cell Ranger pipeline v.3.0.2 (10 x Genomics). Downstream analysis and visualization were done using Seurat (version 4.0.5) implemented in R (version 4.1.1). After inspection of the quality control metrics, cells with > 15% of mitochondrial content or <2500 detected genes were excluded from the downstream analysis. We normalized and scaled the unique molecular identifier (UMI) counts using regularized negative binomial regression. Afterward, we performed linear dimensionality reduction (principal component analysis) and used the top 20 principal components to perform the unsupervised Uniform Manifold Approximation and Projection (UMAP) and clustering, which were computed at a range of resolutions from 1.2 to 0.05. The let-7a-5p high and low imMKCL populations were combined for all subsequent analyses. Cell clusters were identified using the FindClusters function from Seurat. Five clusters for DOX-ON samples were identified (resolution 0.2). The FindAllMarkers function implemented in Seurat was used to identify DEGs between different clusters. The Wilcoxon test was performed on each gene, and the *P*-values and adjusted *P*-values for statistical significance were computed. Genes with adjusted *P*-values <0.01 were considered significant. The g:Profiler was used for the identification of enriched functional terms from the GO.

### Statistical analysis

Statistical analysis was performed using GraphPad Prism software (GraphPad Software, La Jolla, CA). Data are expressed as the mean standard error of the mean (SEM). Values of *P* < 0.05 were considered significant. Details of the sample size used, statistics, and statistical significance are indicated in each figure legend.

### Reporting summary

Further information on research design is available in the Nature Portfolio Reporting Summary linked to this article.

### Supplementary information

Supplementary Information

Peer Review File

Reporting Summary

### Source data

Source Data

## Data Availability

The raw sequence data have been deposited in the DNA Data Bank of Japan (DDBJ) database under the association number PRJDB15883, composing of the following subseries: DRA016355, DRA016356, DRA016432. The project is also available at the NCBI BioProject [http://www.ncbi.nlm.nih.gov/bioproject/] under the same association number. The processed data sets were deposited with the association codes E-GEAD-615, E-GEAD-616, E-GEAD-617. Source data are provided with this paper. The authors declare that all data supporting the findings of this study are available within the article and its Supplementary Information file. Source data are provided with this paper.
