# Supplementary material for: A let-7 microRNA-RALB axis links the immune properties of iPSC-derived megakaryocytes with platelet producibility
Source: Nat Commun. 2024 Mar 22;15:2588. doi: 10.1038/s41467-024-46605-0 (PMC10960040; doi:10.1038/s41467-024-46605-0)
Supplement: Supplementary file 1 — Supplementary Information [file 41467_2024_46605_MOESM1_ESM.pdf]

Supplementary Information

A let-7 microRNA-RALB axis links the immune properties of iPSC-derived megakaryocytes with platelet producibility

Si Jing Chen, Kazuya Hashimoto, Kosuke Fujio, Karin Hayashi, Sudip Kumar Paul, Akinori Yuzuriha, Wei-Yin Qiu, Emiri Nakamura, Maria Alejandra Kanashiro, Mio Kabata, Sou Nakamura, Naoshi Sugimoto, Atsushi Kaneda, Takuya Yamamoto, Hirohide Saito, Naoya Takayama, Koji Eto

Supplementary Methods

Differentiation of $CD34^+$ cells from human ESCs

The human ESC line KhES-3 was obtained from the Institute for Frontier Medical Sciences, Kyoto University (Kyoto, Japan), and maintained in AK02N medium (Ajinomoto, Tokyo, Japan) on iMatrix-511 silk (Takara Bio, Shiga, Japan)-precoated 6-well plates. $CD34^+$ HPC differentiation was performed using our previously established Sac method[1, 2]. Briefly, small clumps of equivalent cell numbers (1.5 to 1.6×10^5 cells) were transferred onto a 10-cm dish coated with mitomycin C-treated C3H10T1/2 mouse feeder cells (Riken Bio-Resource Center, Tsukuba, Ibaraki, Japan) and cultured in basal medium (IMDM, Sigma-Aldrich, St. Louis, MO, USA) supplemented with L-glutamine (Thermo Fisher Scientific, Waltham, MA, USA), insulin-transferrin-selenium (Thermo Fisher Scientific), 50 μg/mL ascorbic acid (Sigma-Aldrich), 450 μM α-monothioglycerol (Sigma-Aldrich), and 20 ng/mL recombinant human VEGF (Wako, Osaka, Japan). Then, 50 ng/mL basic FGF (Wako) and 10 μM SB431542 (Wako) were added on days 4 to 7, and 10 U/mL Heparin (AY pharmaceuticals, Tokyo, Japan) on days 4 to 10. Medium changes were performed on days 4, 7, and 11 (Supplementary Fig. 2a). On day 14, the cells were collected with a cell scraper and filtered with a cell strainer for further investigation.

Purification of human cord blood-derived $CD34^+$ cells

Fresh umbilical cord blood (UCB) samples were obtained from healthy donors sourced from the Japanese Red Cross Society Kanto-Koshinetsu Blook Blood Center. Mononuclear cells (MNC) were isolated through density centrifugation using lymphocyte separation medium (Ficoll). The cells were subsequently incubated with the immunomagnetic beads (CD34) (Miltenyi Biotech) and selected using a QuadroMACS® separator according to manufacturer's instructions. The efficiency for the purification was verified by flow cytometry. The resulting $CD34^+$ fraction was aliquoted and stored in liquid nitrogen until use.

Differentiation of megakaryocytes from cord blood-derived $CD34^+$ cells

The *in vitro* differentiation of cord blood-derived $CD34^+$ cells was performed in accordance with a previously published protocol with minor modifications[3, 4]. Briefly, $CD34^+$ cells were expanded in X-VIVO 10 media (Lonza), supplemented with 1% bovine serum albumin (BSA, StemCell Technologies), 100 ng/mL human SCF (R&D Systems), 100 ng/mL human Flt-3 ligand (PeproTech EC Ltd.), 50 ng/mL human thrombopoietin (TPO, R&D Systems), 10 ng/mL human IL-6 (PeproTech EC Ltd.), and 10 ng/mL human IL-3 (PeproTech EC Ltd.). Following a 9-day culture period, the cells were harvested and counted. 4×10^5 cells were seeded and cultured for an additional 11 days in a total volume of 1 mL X-VIVO 10 media supplemented with 1% BSA, SCF, TPO, and IL-6. RNA isolation was isolated on day 16, and flow cytometry analysis was

conducted on day 20 to evaluate $CD41a^{+}CD42b^{+}$ platelet-like particles. The media was changed every two days across the differentiation period (Supplementary Fig. 11a).

siRNA-mediated gene knockdown

The Dharmacon™ siGENOME siRNA SMARTpool designed against human RALB (siRALB), which is comprised of four siRNAs targeting different sites, as well as a non-targeting control siRNA (siNT) were purchased from Horizon Discovery Ltd. The gene silencing procedure was performed based on the Dharmacon reverse transfection protocol with minor modification. Briefly, siRNA at 10 nM final concentration was diluted in transfection buffer from the Stemfect RNA Transfection Kit (ReproCELL), and transfection reagent was diluted in an equal volume of buffer. The diluted siRNA and transfection reagent were combined and incubated at room temperature for 15 min. Subsequently, 12.5 μL/well of transfection mixture was added to the wells of a 96-well plate, followed by the addition of 2×10^{5} cells in 100 μL medium to each well. After a 4-hour incubation period, the cells were washed with PBS and subjected to either proliferation or differentiation culture conditions. To validate the gene knockdown efficiency, total RNA was isolated and analyzed 48 hours after the transfection.

Addition of recombinant human interferon-α2a or IL-8 in imMKCL culture

To examine the potential involvement of interferons or IL-8 in the proliferation and iPSC-PLT production of imMKCLs, cells were cultured under either proliferation or differentiation culture conditions with the addition of recombinant human interferon-α2a (Miltenyi Biotec) or IL-8 (R&D systems). Concentrations ranging from 0 to 100 ng/mL were utilized as working concentrations. The proliferation and iPSC-PLT production capacity of imMKCLs were analyzed accordingly.

Addition of small molecules in imMKCL culture

The impact of two small molecules on the proliferation and iPSC-PLT production of imMKCLs was investigated: (1) Reparixin (Selleckchem), a specific inhibitor of CXCL8 receptor CXCR1/2 that is commonly used to inhibit the IL-8 signaling pathway; and (2) RBC8 (Selleckchem), a selective inhibitor of the GTPases RALA and RALB. imMKCLs were cultured under either proliferation or differentiation culture conditions in the absence or presence of Reparixin or RBC8 at working concentrations ranging from 0 to 10 μM. For these studies, dimethyl sulfoxide (DMSO) was added in control wells. Notably, the application of a high concentration (10 μM) of either molecule arrested the proliferation.

Intracellular flow cytometry

The intracellular flow cytometry assay was optimized for imMKCLs as follows. Cells were fixed in 4% paraformaldehyde (PFA) for 15 mins at room temperature, followed by methanol permeabilization (15 mins on ice). After two washes with PBS, the cells were incubated with anti-RalB antibody (Merck Millipore, 1:3000) for 1 hour on ice. Subsequently, the cells were washed and incubated with a goat anti-mouse secondary antibody, Alexa Fluor™ 647 (Invitrogen, 1:1000) on ice. The stained cells and non-staining controls were then analyzed by flow cytometry.

PAC-1 binding and *P*-selectin expression of iPSC-PLTs

The measurement of PAC-1 binding and *P*-selectin expression levels adhered to the protocol outlined in our previous study[5]. Briefly, the culture suspensions (iPSC-PLTs) were stimulated with or without phorbol-12-myristate-13-acetate (PMA, 0.2 μM) or adenosine triphosphate plus thrombin receptor activator peptide 6 (ADP/TRAP6, 100 μM and 40 μM, respectively). Subsequently, the mixtures were incubated with BV421 mouse anti-human CD62P (BD Biosciences), APC-anti-human CD41a (Bio Legend), and FITC-PAC-1 (BD Biosciences) antibodies. Following a 30-min incubation at room temperature, the samples were diluted with Hepes-Tyrode buffer and analyzed by flow cytometry.

References

1. Takayama, N. *et al.* Generation of functional platelets from human embryonic stem cells in vitro via ES-sacs, VEGF-promoted structures that concentrate hematopoietic progenitors. *Blood* **111**, 5298-5306 (2008).
2. Yuzuriha, A. *et al.* Extracellular laminin regulates hematopoietic potential of pluripotent stem cells through integrin β1-ILK-β-catenin-JUN axis. *Stem Cell Res* **53**, 102287 (2021).
3. Kaushansky, K. *et al.* Thrombopoietin, the Mpl ligand, is essential for full megakaryocyte development. *Proc Natl Acad Sci U S A* **92**, 3234-3238 (1995).
4. Bruno, S. *et al.* In vitro and in vivo megakaryocyte differentiation of fresh and ex-vivo expanded cord blood cells: rapid and transient megakaryocyte reconstitution. *Haematologica* **88**, 379-387 (2003).
5. Sugimoto, N. *et al.* Production and nonclinical evaluation of an autologous iPSC-derived platelet product for the iPLAT1 clinical trial. *Blood Adv* **6**, 6056-6069 (2022).

Supplementary Table 1 The target miRNAs included in the miRNA switch screening library.

miR-1234-5p	miR-185-3p	miR-301a-5p	miR-425-3p	miR-497-5p	miR-10b-3p
miR-329	miR-144-3p	miR-211-3p	miR-485-5p	miR-450-5p	miR-379-5p
miR-629-5p	miR-362-3p	miR-589-5p	miR-671-5p	miR-506-3p	miR-125b-1-3p
miR-30b-3p	miR-296-3p	miR-506-5p	miR-361-3p	miR-582-3p	miR-422a
miR-98-3p	miR-499a-5p	miR-24-2-5p	miR-105-5p	miR-381-3p	miR-181b-3p
miR-122-5p	miR-27a-5p	miR-505-5p	miR-454-3p	miR-301a-3p	miR-510
miR-411-5p	miR-29c-5p	miR-148b-5p	miR-204-3p	miR-486-3p	miR-125b-2-3p
miR-212-5p	miR-582-5p	miR-197-5p	miR-222-5p	miR-93-3p	miR-483-5p
miR-576-5p	miR-146a-3p	miR-548-3p	miR-34a-3p	miR-190a	miR-383
miR-140-5p	miR-326	miR-576-3p	miR-219-5p	miR-342-5p	miR-590-3p
miR-133a	miR-17-3p	miR-17-5p	miR-1	miR-206	miR-21-5p
miR-367-3p	miR-373-5p	miR-92a-3p	miR-16-5p	miR-197-3p	miR-24-3p
miR-339-5p	miR-224-5p	miR-127-3p	miR-365a-3p	miR-183-5p	miR-331-3p
miR-203a	miR-214-3p	miR-137	miR-298	miR-325	miR-449a
miR-498	miR-661	miR-429	miR-1182	miR-208a-3p	miR-320b
miR-423-5p	miR-140-3p	miR-378c	miR-185-5p	miR-101-3p	miR-378b
miR-128	miR-509-5p	miR-340-5p	miR-25-5p	miR-92b-3p	miR-320c
miR-508-3p	miR-744-5p	miR-3180-3p	miR-30d-3p	miR-215	miR-4510
miR-1260a	miR-151a-5p	miR-584-5p	miR-503-5p	miR-34c-5p	miR-3180
miR-514a-3p	miR-374b-5p	miR-28-3p	miR-1307-5p	miR-21-3p	miR-193a-5p
miR-1269a	miR-365b-3p	miR-4286	miR-4454	miR-138-5p	miR-1307-3p
miR-652-3p	miR-502-3p	miR-92b-5p	miR-501-3p	miR-1285-3p	miR-126-3p

miR-4531	miR-193b-5p	miR-181a-2-3p	miR-1301	miR-877-5p	miR-4443
miR-425-5p	miR-320d	let-7a-3p	miR-92a-1-5p	miR-365b-5p	miR-142-3p
miR-320e	miR-106b-3p	miR-548	miR-1261	miR-9-3p	miR-129-1-3p
miR-424-3p	miR-760	miR-365a-5p	miR-374a-5p	miR-873-5p	miR-30c-2-3p
miR-99b-3p	miR-4532	20b-3p	miR-4448	miR-363-5p	miR-145-3p
miR-574-5p	miR-223-3p	miR-4521	miR-22-5p	miR-339-3p	miR-16-2-3p
miR-374a-3p	miR-542-3p	miR-452-5p	miR-513a-3p	miR-532-39	miR-548a-3p
miR-27b-5p	miR-3180-5p	miR-4324	let-7d-3p	miR-184	miR-95
miR-664a-5p	let-7a-5p	let-7g-5p	let-7i-5p	miR-191-5p	miR-125b-5p
miR-100-5p	miR-342-3p	miR-210	miR-193b-3p	miR-99b-5p	miR-106b-5p
miR-27a-3p	miR-30a-3p	miR-423-3p	miR-409-3p	miR-186-5p	miR-324-3p
miR-22-3p	miR-378a-3p	miR-196b-5p	miR-296-5p	miR-99a-5p	miR-34a-5p
miR-501-5p	miR-500a-3p	miR-155-5p	miR-182-5p	miR-10a-5p	miR-345-5p
miR-335-5p	miR-362-5p	miR-193a-5p	miR-134	miR-195-5p	miR-26a-5p
miR-151a-3p	miR-200c-3p	miR-212-3p	miR-143-3p	miR-149-5p	miR-28-5p
miR-505-3p	miR-192-5p	miR-187-3p	miR-129-5p	miR-106a-5p	miR-299-3p
miR-483-3p	miR-18a-3p	miR-330-3p	miR-200b-3p	miR-154-5p	miR-150-5p
miR-145-5p	miR-199a-5p	miR-135a-5p	miR-200a-3p	miR-133b	miR-141-3p
miR-148a-3p	miR-9-5p	miR-520c-3p	miR-328	miR-361-5p	miR-375
miR-378a-5p	miR-382-5p	miR-484	miR-486-5p	miR-7-5p	miR-124-3p
miR-302a-5p	miR-370	miR-373-3p	miR-492	miR-509-3p	miR-512-5p
miR-516b-5p	miR-518b	miR-518c-3p	miR-518c-5p	miR-519d	miR-520f
miR-520g	miR-523-3p	miR-525-5p	miR-526a	miR-98-5p	

Supplementary Table 2 Significantly enriched immune-related gene sets in let-7 low imMKCLs at the proliferation or differentiation stages and in let-7 low ESC-derived $CD34^{+}$ cells. The normalized enrichment score (NES), the determined nominal (non-adjusted) *p* value, and the false discovery rate (FDR) were derived from GSEA software.

GSEA Gene Sets	NES	*P* value	FDR
**imMKCLs (Dox-ON)**			
Immune response to enterotoxin	2.471	0.000	0.000
TNF signaling via NFKB	2.295	0.000	0.000
NFKB targets	2.172	0.000	0.000
IFNB1 targets	2.304	0.000	1.54E-04
Inflammatory response to LPS	2.279	0.000	2.42E-04
Interferon responsive genes	2.215	0.000	0.003
**imMKCLs (Dox-OFF)**			
Immune response to enterotoxin	2.665	0.000	3.41E-04
TNF signaling via NFKB	2.578	0.000	0.000
NFKB targets	2.214	0.000	0.008
IFNB1 targets	2.843	0.000	0.000
Inflammatory response to LPS	2.041	0.000	0.002
Interferon responsive genes	2.931	0.000	0.000
**ESC-derived CD34+ HPCs**			
Immune response to enterotoxin	1.926	0.000	0.005
TNF signaling via NFKB	1.870	0.000	0.009
NFKB targets	1.719	0.003	0.032
IFNB1 targets	2.317	0.000	0.000
Inflammatory response to LPS	1.665	0.000	0.045
Interferon responsive genes	2.408	0.000	0.000

Supplementary Table 3 Primer pairs used in RT-qPCR.

Gene symbol	Primer	Sequences（5'- 3'）
**ISG15**	Forward	GAGAGGCAGCGAACTCATCT
	Reverse	CTTCAGCTCTGACACCGACA
**CCL5**	Forward	ATCCTCATTGCTACTGCCCTC
	Reverse	GCCACTGGTGTAGAAATACTCC
**IFIT3**	Forward	GAAGGAACTGGGCCGCCTGCTAAG
	Reverse	GCCCTGGCCCATTTCCTCACTACC
**PF4**	Forward	GTGTGAAGACCACCTCCCAG
	Reverse	GCTTGCAGGTCCAAGCAAAT
**PPBP**	Forward	TTGTAGGCAGCAACTCACCC
	Reverse	TGCAAGGCATGAAGTGGTCT
**RALB**	Forward	GACTACGCAGCCATTCGAGA
	Reverse	CCGCTCCTCTAGGTCAGACT
**CUX1**	Forward	ACAGACACTGAAGAACCAAGCCGA
	Reverse	TGTGAGGCCAGCTGGAGGGA
**IRF7**	Forward	CTTCCAAGAAGAGCTGGTGGAAT
	Reverse	TTCCAGCTTCACCAGGAC
**CDKN2A**	Forward	GCCCAACGCACCGAATAGTT
	Reverse	AATCGGGGATGTCTGAGGGA
**GAPDH**	Forward	GTTGCCATCAATGACCCCTTC
	Reverse	CATTGATGACAAGCTTCCCG

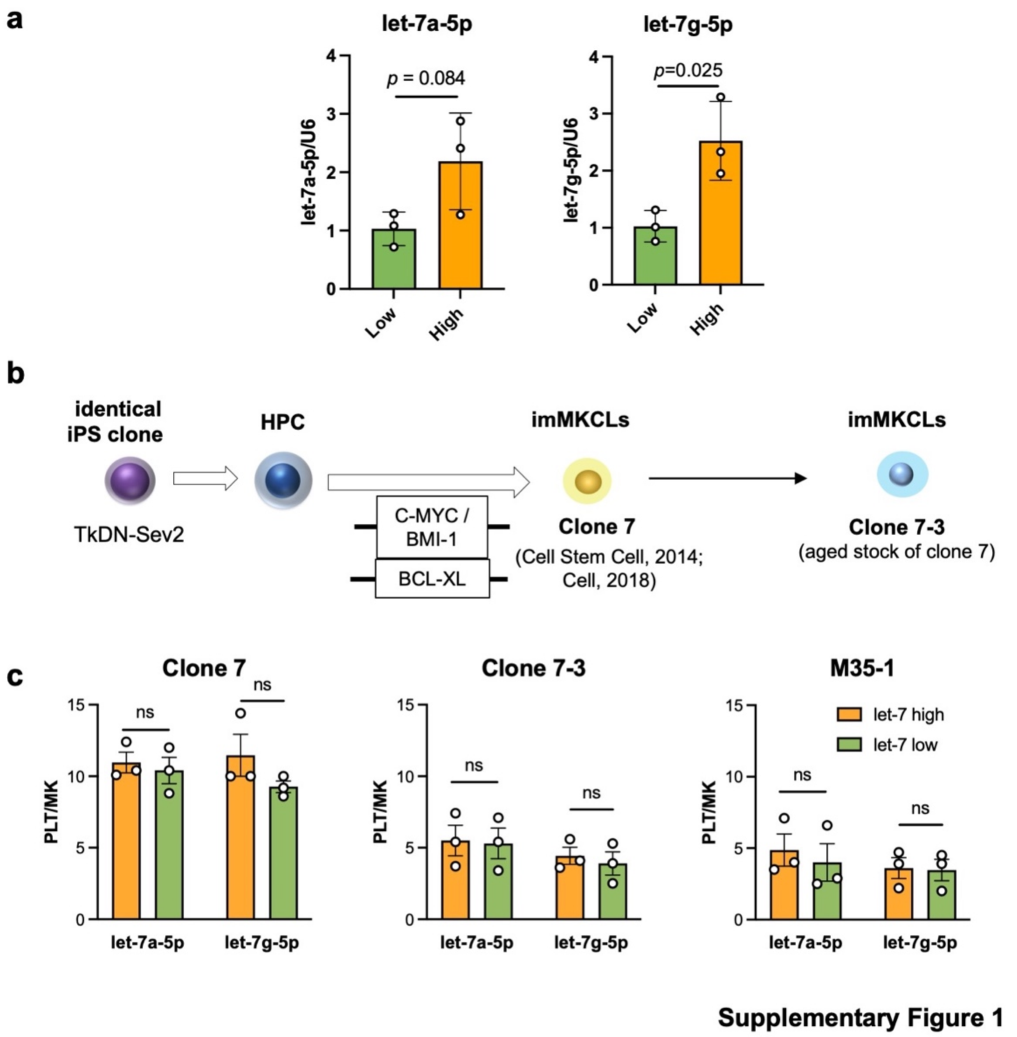

Supplementary Fig. 1

(a) let-7a-5p and let-7g-5p expression levels were determined in let-7 high/low imMKCLs (clone 7) by RT-qPCR. The expression levels were normalized to RNU6B (U6). Sampling was performed 48 hours after sorting. Data are expressed as the mean ± SEM from three independent experiments. **(b)** Clone 7-3 represents an aged stock of clone 7 that shares the same genetic background. **(c)** iPSC-PLT production from let-7 high/low imMKCLs in static conditions. The cells were proceeded to the DOX-OFF step directly after sorting. Three distinct imMKCL clones were employed. Data are expressed as the mean ± SEM from three independent experiments. Unpaired two-tailed student's *t*-tests were used to assess statistical significance. Source data are provided as a Source Data file.

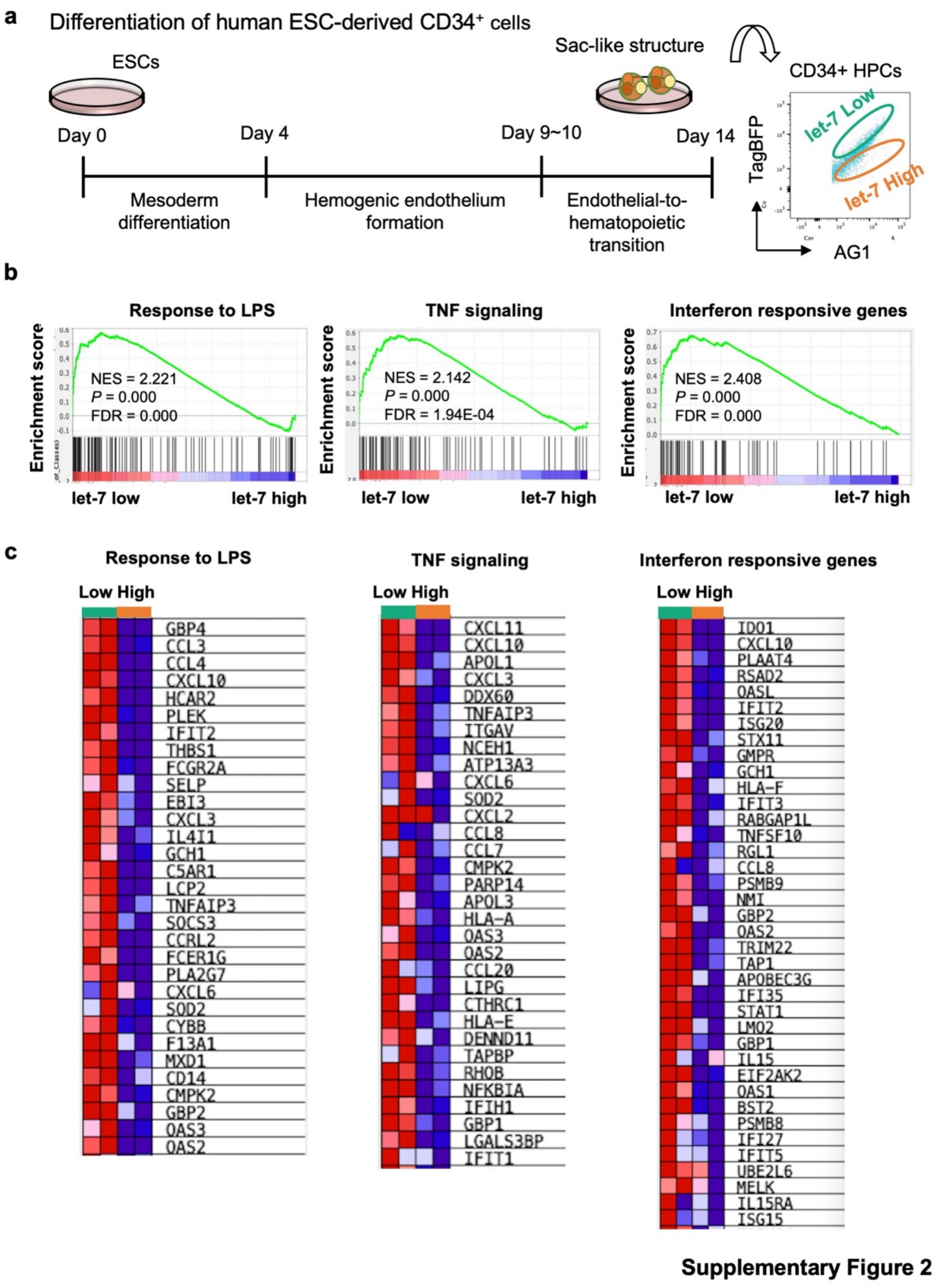

Supplementary Fig. 2

In vitro ESC-derived CD34^{+} HPCs exhibited immune-skewed transcriptional signatures in let-7 low subpopulations. **(a)** A schematic illustration of the ESC-derived HPC induction by the Sac method. **(b)** GSEA plots showing the significantly enriched immune-related gene sets in let-7 low ESC-derived HPCs. **(c)** Heatmaps of the gene sets enriched in the same cells.

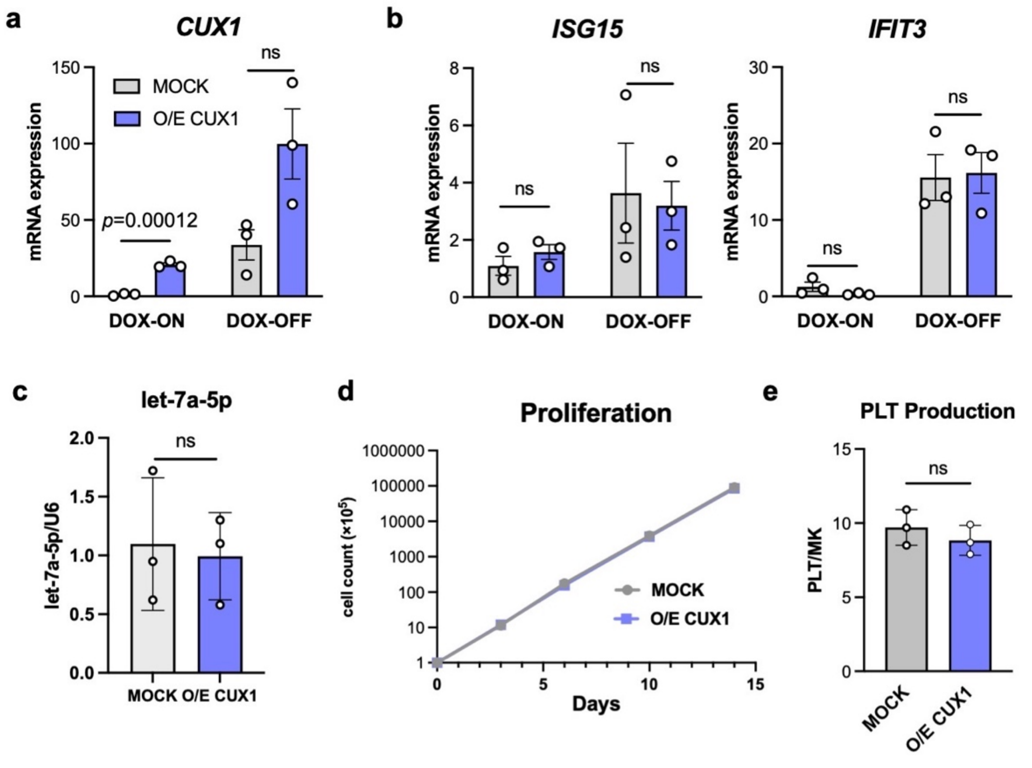

Supplementary Figure 3

Supplementary Fig. 3

(a) The lentiviral-mediated overexpression (O/E) of *CUX1* elevated the mRNA expression of *CUX1* at both the proliferation (DOX-ON) and maturation (DOX-OFF) stages in imMKCLs (clone 7). **(b)** CUX1 overexpression did not affect the mRNA expressions of *ISG15* or *IFIT3*. The expression levels were normalized to *GAPDH*. **(c)** The expression of let-7a-5p was not significantly affected by the overexpression of *CUX1*. The expression levels were normalized to RNU6B. Neither the proliferation rate **(d)** nor the iPSC-PLT production **(e)** in static conditions were affected by CUX1 overexpression. Data are expressed as the mean ± SEM from three independent experiments. Unpaired two-tailed student's *t*-tests were used to assess statistical significance. Source data are provided as a Source Data file.

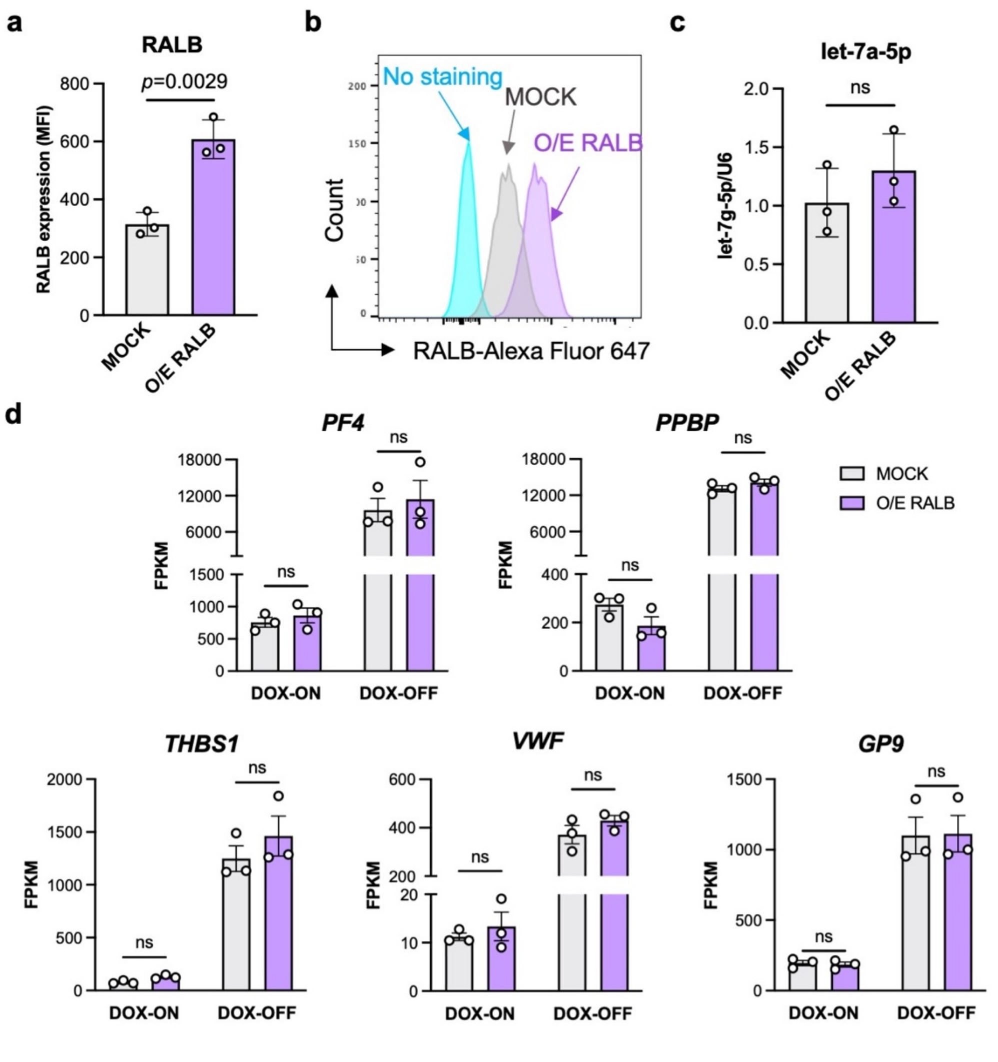

Supplementary Figure 4

Supplementary Fig. 4

(a) The intracellular protein expression of RALB (mean fluorescence intensity, MFI) detected in MOCK or RALB-overexpressing imMKCLs (O/E RALB) by intracellular flow cytometry. **(b)** The RALB expression was compared using a histogram overlay. **(c)** Comparable let-7a-5p expression was observed in MOCK and RALB-overexpressing imMKCLs. **(d)** The overexpression of RALB did not significantly impact the expression of genes related to MK maturation. The bar graphs show FPKM values of the indicated genes. Data are expressed as the mean ± SEM from three independent experiments. Unpaired two-tailed student's t-tests were used to assess statistical significance. Source data are provided as a Source Data file.

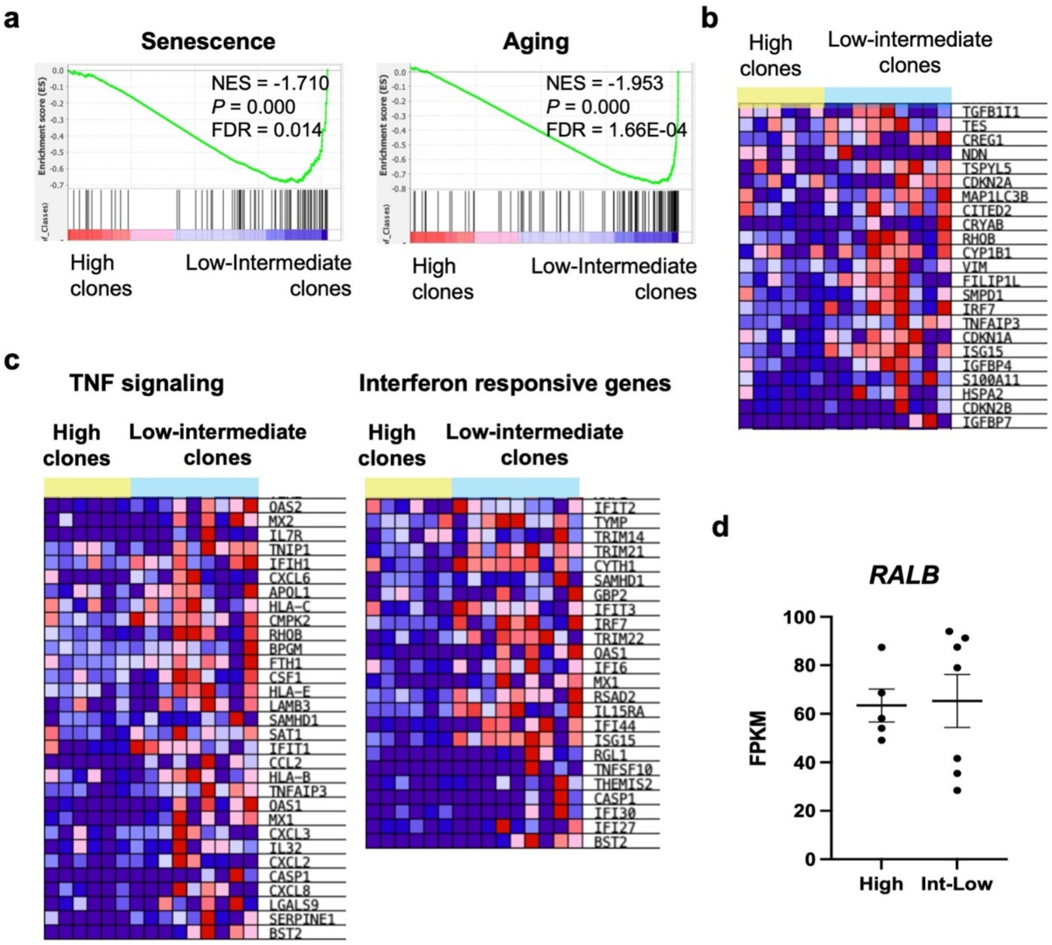

Supplementary Figure 5

Supplementary Fig. 5

GSEA were performed to compare high quality clones (n=6) and Low ~ intermediate clones (n=9). **(a)** GSEA plots and **(b)** a heatmap showing the enrichment of senescence and aging-related gene sets in low~intermediate quality clones compared with high quality clones. Representative enrichment plots from each group are displayed with the NES, non-adjusted *p* value, and FDR derived from GSEA software. **(c)** Heatmaps of immune-related gene sets enriched in low~intermediate quality clones. **(d)** *RALB* expression levels in clones with distinct qualities. Source data are provided as a Source Data file.

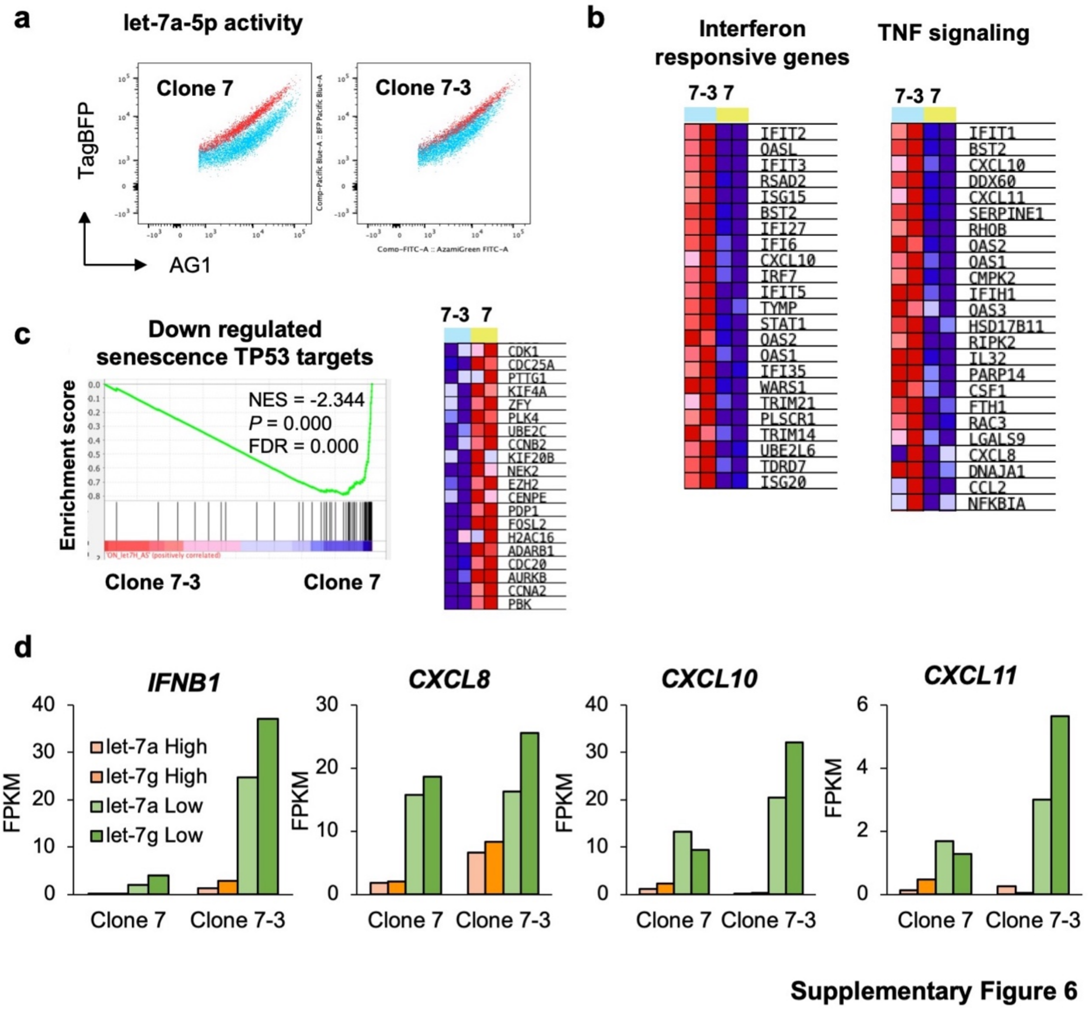

Supplementary Fig. 6

(a) A flow cytometry analysis of let-7a-5p activity patterns in clone 7 and clone 7-3 by miRNA switch technology. **(b)** Heatmaps of gene sets enriched in clone 7-3. The let-7 low cells from each clone were compared. **(c)** GSEA plots showing the enrichment of downregulated senescence TP53 targets in clone 7-3 compared with clone 7. **(d)** Inflammatory cytokine encoding genes exhibited increased expression levels in clone 7-3 compared with clone 7. Source data are provided as a Source Data file.

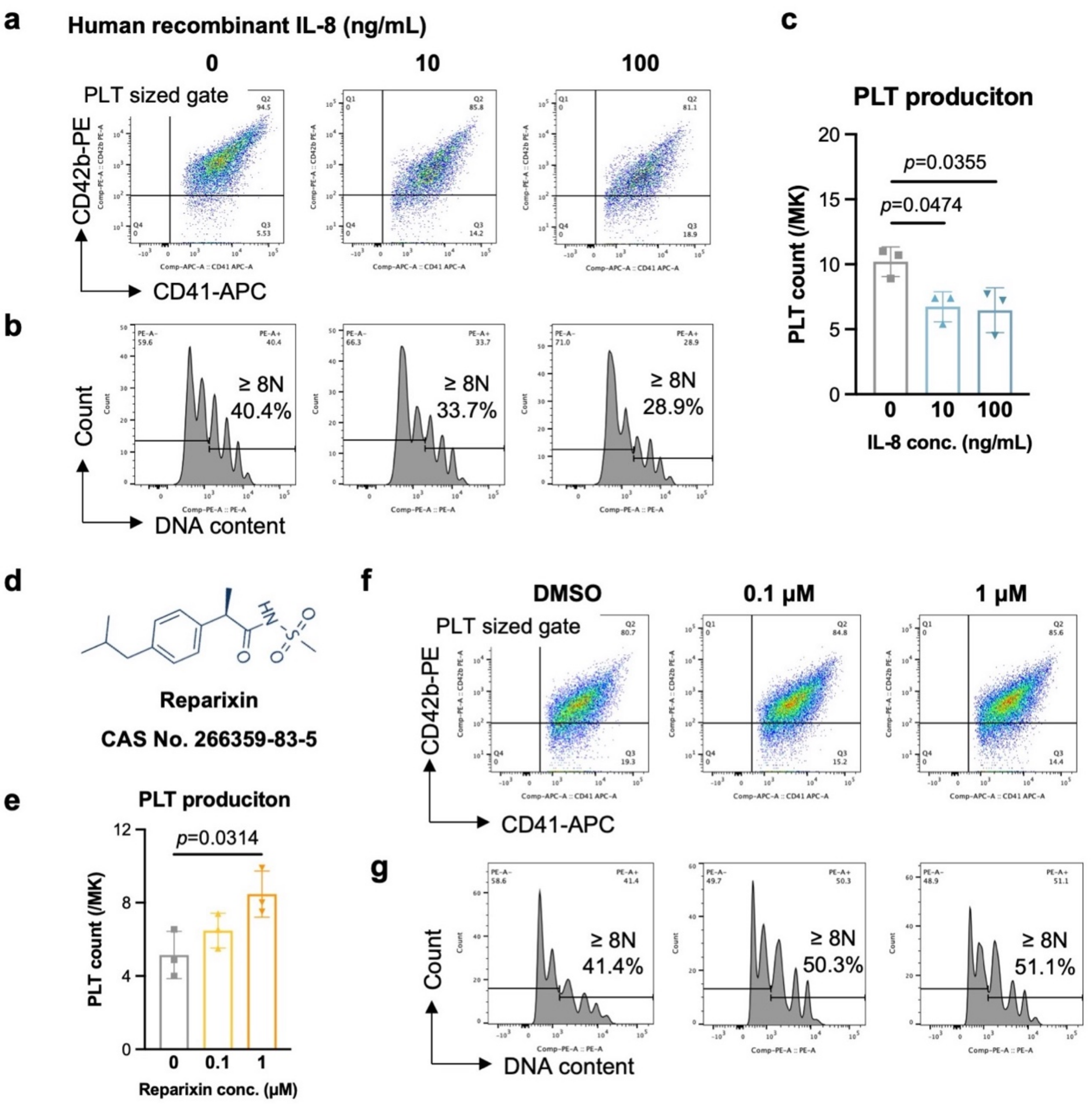

Supplementary Figure 7

Supplementary Fig. 7

(a) Representative flow cytometry plots of iPSC-PLTs generated from clone 7 in the presence of human recombinant IL-8 at the indicated concentrations in static conditions. **(b)** Flow cytometric analysis of ploidy. **(c)** iPSC-PLT production under the indicated conditions. **(d)** Molecular structure of Reparixin, a specific inhibitor of CXCR1/2. **(e)** iPSC-PLT production under the indicated conditions. **(f)** Representative flow cytometry plots of iPSC-PLTs generated from RALB-overexpressing imMKCLs (clone 7) in the presence of Reparixin at the indicated concentrations. **(g)** Flow cytometric analysis of ploidy. Data are expressed as the mean ± SD from three independent experiments. One-way ANOVA with multiple comparisons was used to assess statistical significance. Source data are provided as a Source Data file.

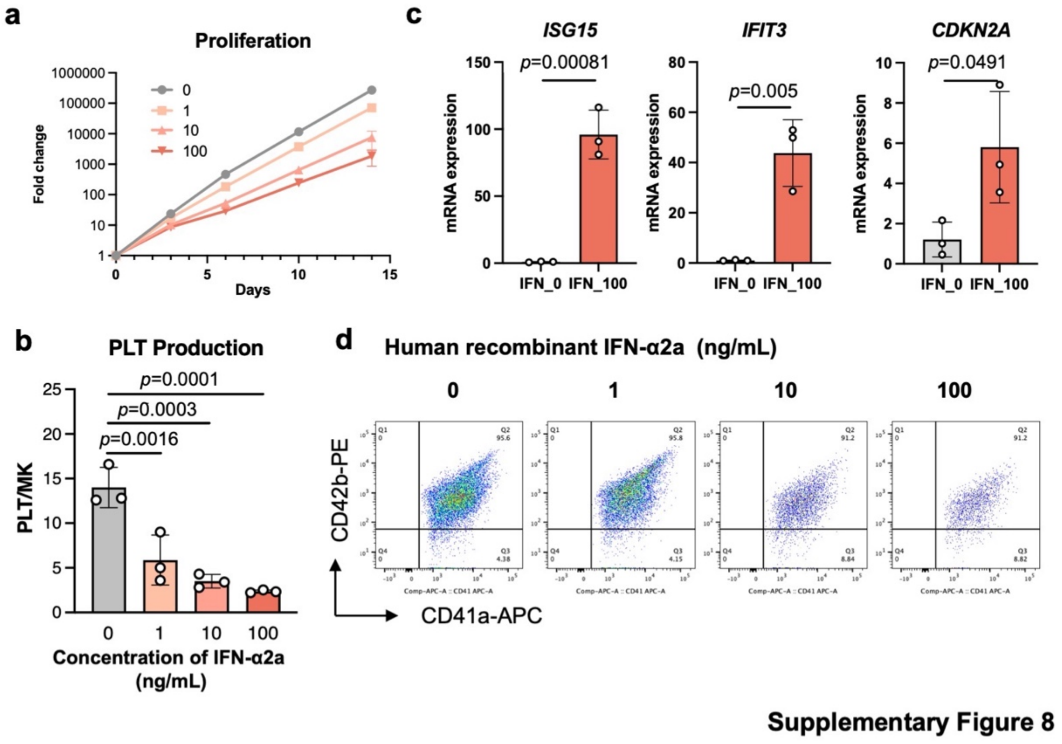

Supplementary Fig. 8

Treatment with human recombinant interferon-α2a inhibited the proliferation **(a)** and iPSC-PLT production **(b)** of imMKCLs in a dose-dependent manner. imMKCLs (clone 7) were treated with human IFN-α2a at concentrations of 0, 1, 10, or 100 ng/mL in static conditions. **(c)** The induced mRNA expression of interferon-related genes and *CDKN2A* in imMKCLs. mRNA expression levels were measured by RT-qPCR and normalized to *GAPDH*. **(d)** Representative flow cytometry plots of iPSC-PLTs generated under the indicated conditions. Data are expressed as the mean ± SD from three independent experiments. One-way ANOVA with multiple comparisons or unpaired two-tailed student t-tests were used to assess statistical significance. Source data are provided as a Source Data file.

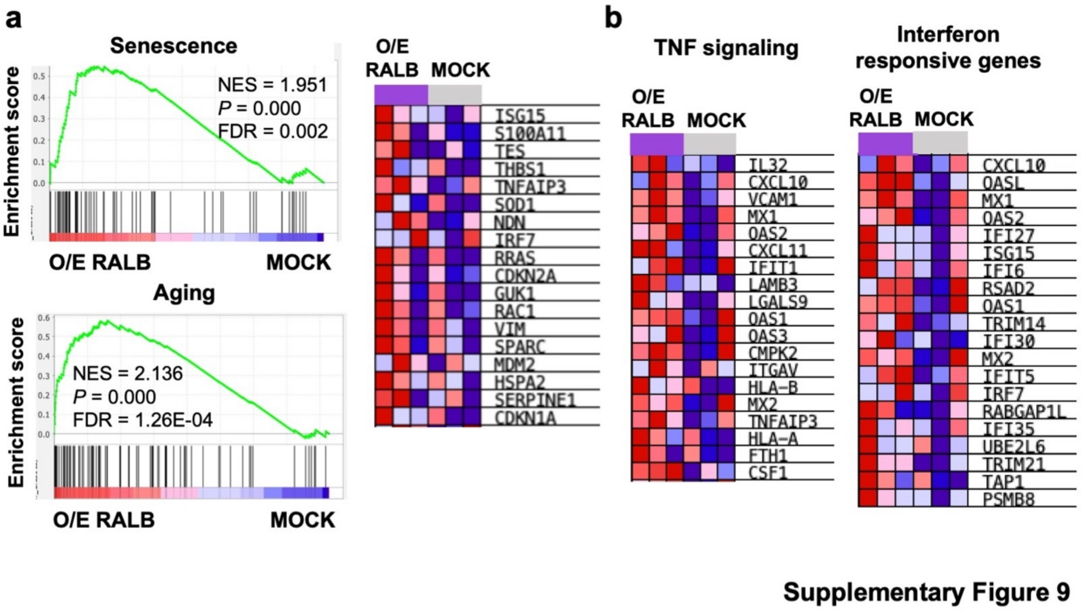

Supplementary Fig. 9

(a) GSEA plots and a heatmap showing the enrichment of senescence and aging-related gene sets in RALB-overexpressing imMKCLs (O/E RALB) compared with MOCK. **(b)** Heatmaps of the gene sets enriched in O/E RALB (clone 7).

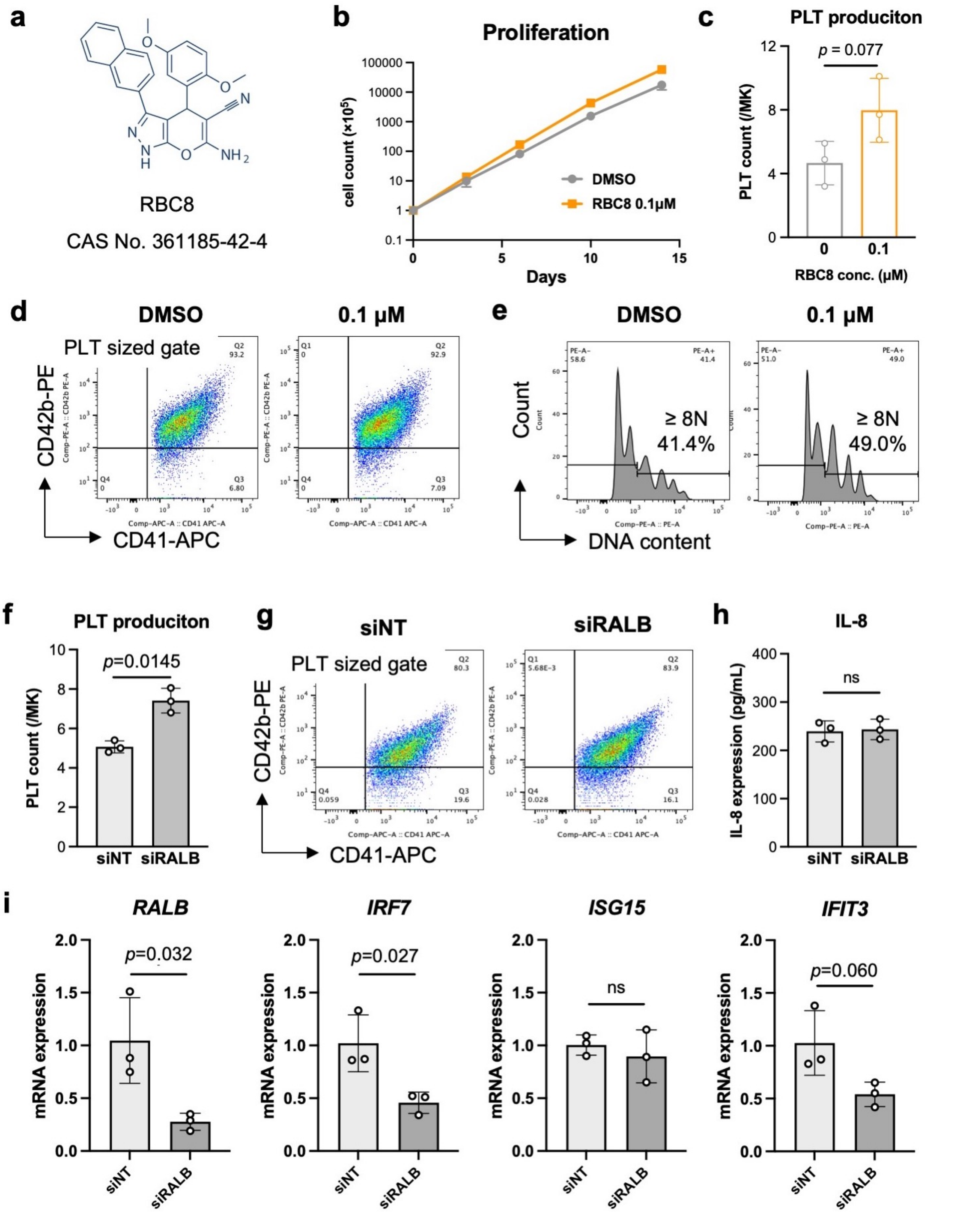

Supplementary Figure 10

Supplementary Fig. 10

(a) Molecular structure of RBC8, a specific inhibitor of RALA and RALB. **(b)** The administration of 0.1 μM RBC8 inhibited the proliferation of imMKCLs (clone 7). **(c)** iPSC-PLT production under the indicated conditions. **(d)** Representative flow cytometry plots of iPSC-PLTs generated

from RALB-overexpressing imMKCLs (clone 7) in the presence or absence of RBC8. **(e)** Flow cytometric analysis of ploidy. **(f)** siRNA-mediated *RALB* knockdown ameliorated the iPSC-PLT production of imMKCLs (clone 7). Cells were transfected with siRNA targeting *RALB* (siRALB) or non-targeting control (siNT). (**g)** Representative flow cytometry plots of iPSC-PLTs generated from siNT or siRALB. **(h)** RALB knockdown did not significantly impact IL-8 secretion at the maturation stage. **(i)** The relative mRNA expression of the indicated genes was assessed in imMKCLs 48 hours following the reverse transfection procedure. mRNA expression levels were measured by RT-qPCR and normalized to *GAPDH*. The iPSC-PLTs were generated in static conditions. Data are expressed as the mean ± SEM from three independent experiments. Unpaired two-tailed student's *t*-tests were used to assess statistical significance. Source data are provided as a Source Data file.

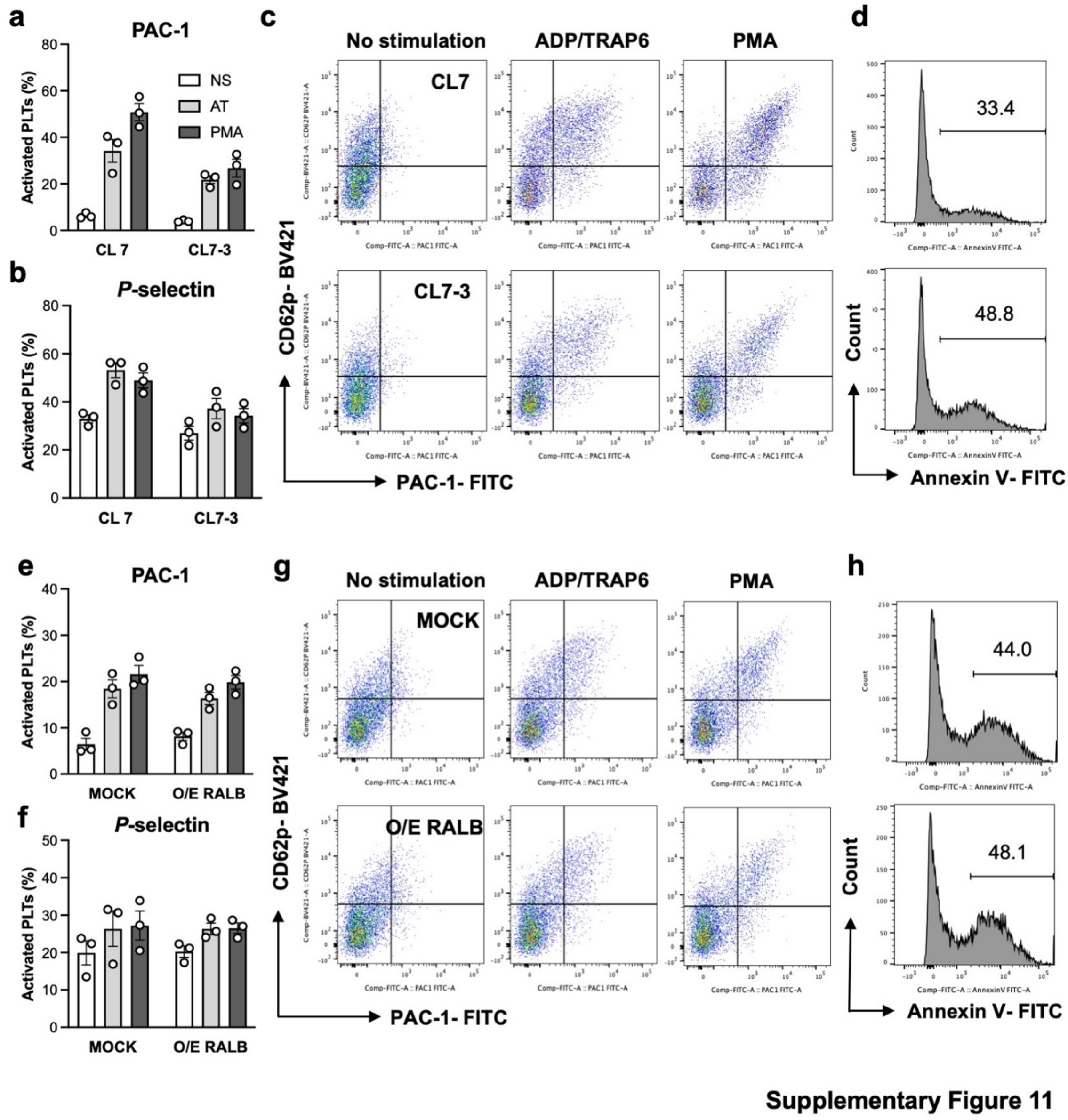

Supplementary Fig. 11

(a) PAC-1 binding and **(b)** *P*-selectin expression on iPSC-PLTs generated from clone 7 or clone 7-3 with or without PMA or ADP/TRAP6 stimulation. **(c)** Representative flow cytometry plots of activated iPSC-PLTs. **(d)** Representative histograms of Annexin V bound to CD41+ iPSC-PLTs derived from clone 7 or clone 7-3. **(e)** PAC-1 binding and **(f)** *P*-selectin expression on iPSC-PLTs generated from MOCK or RALB-overexpressing imMKCLs (O/E RALB) with or without PMA or ADP/TRAP6 stimulation. **(g)** Representative flow cytometry plots of activated iPSC-PLTs. **(h)** Representative histograms of Annexin V bound to CD41+ iPSC-PLTs derived from MOCK or O/E RALB. The iPSC-PLTs were generated in turbulence conditions. Data are expressed as the mean ± SEM from three independent experiments. Source data are provided as a Source Data file.

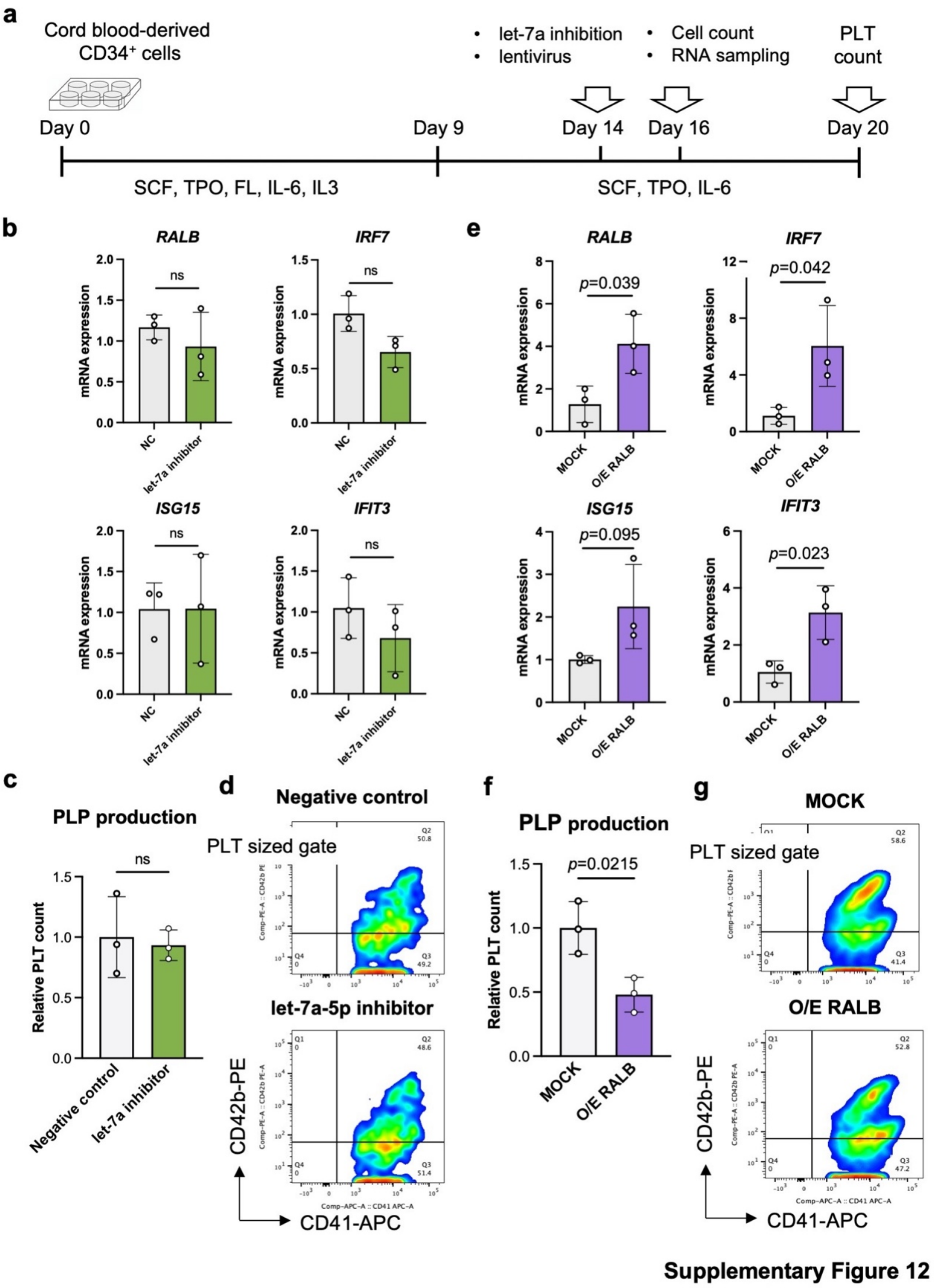

Supplementary Fig. 12

(a) A schematic illustration of the *in vitro* differentiation of MKs from cord blood-derived CD34^{+} cells. The inhibition of let-7a-5p significantly impacted neither mRNA expression of the indicated

genes **(b)** nor platelet-like particles (PLP) production **(c-d)**. **(e)** Significantly elevated expression levels of *RALB*, *IRF7, ISG15*, and *IFIT3* were observed in RALB-overexpressing (O/E) cells. Total RNAs were isolated from day-16 cells. The mRNA expression levels were measured by RT-qPCR and normalized to *GAPDH*. **(f-g)** The overexpression of RALB induced a significant decline of CD41a^{+}CD42b^{+} PLPs generated from cord blood-derived MKs at day 20. Data are expressed as the mean ± SEM from three independent experiments. Unpaired two-tailed student's *t*-tests were used to assess statistical significance. Source data are provided as a Source Data file.

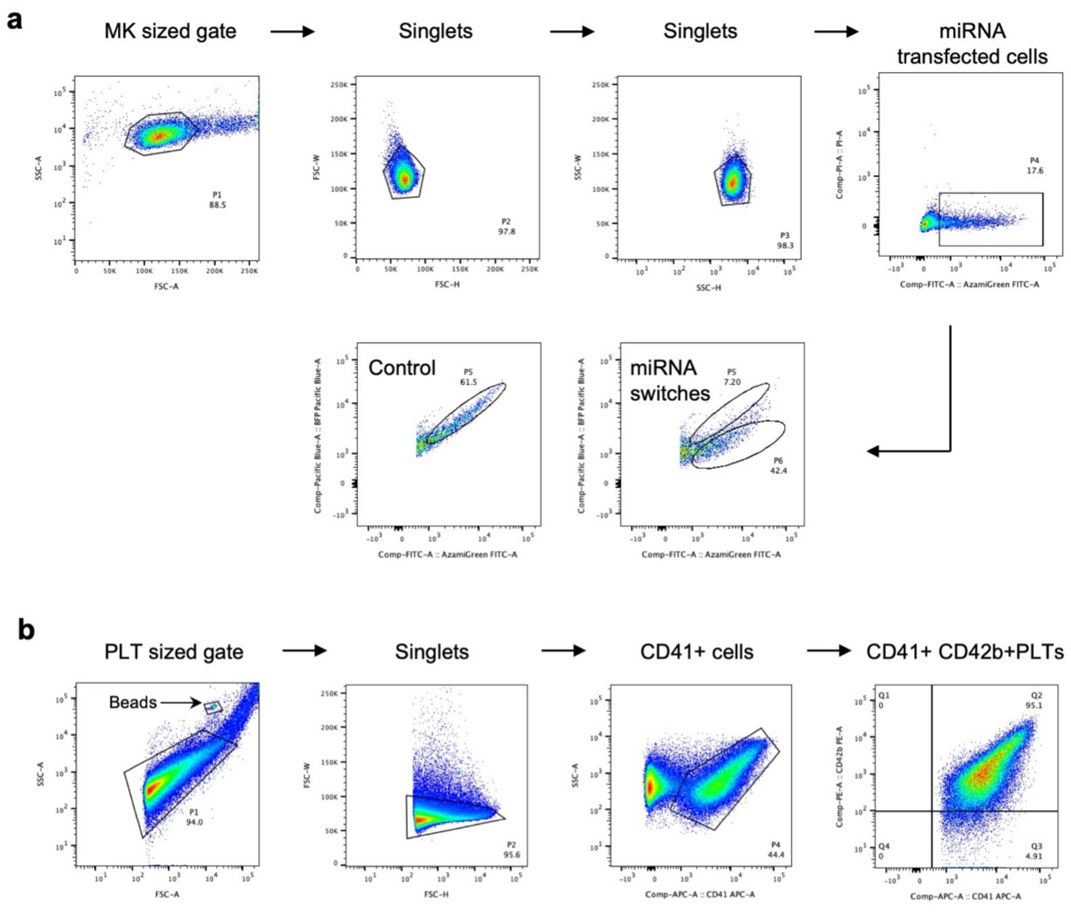

Supplementary Figure 13

Supplementary Fig. 13

Flow cytometry gating strategies for imMKCL subpopulations distinguished by miRNA switches **(a)** and for iPSC-PLTs counted with the aid of absolute counting beads **(b)**.
