# Supplementary material for: A let-7 microRNA-RALB axis links the immune properties of iPSC-derived megakaryocytes with platelet producibility
Source: Nat Commun. 2024 Mar 22;15:2588. doi: 10.1038/s41467-024-46605-0 (PMC10960040; doi:10.1038/s41467-024-46605-0)
Supplement: Supplementary file 3 — Reporting Summary [file 41467_2024_46605_MOESM3_ESM.pdf]

nature portfolio

Corresponding author(s): Koji Eto

Last updated by author(s): Feb 29, 2024

Reporting Summary

Nature Portfolio wishes to improve the reproducibility of the work that we publish. This form provides structure for consistency and transparency in reporting. For further information on Nature Portfolio policies, see our Editorial Policies and the Editorial Policy Checklist.

Statistics

For all statistical analyses, confirm that the following items are present in the figure legend, table legend, main text, or Methods section.

n/a	Confirmed	
☐	☒	The exact sample size (*n*) for each experimental group/condition, given as a discrete number and unit of measurement
☐	☒	A statement on whether measurements were taken from distinct samples or whether the same sample was measured repeatedly
☐	☒	The statistical test(s) used AND whether they are one- or two-sided<br>*Only common tests should be described solely by name; describe more complex techniques in the Methods section.*
☒	☐	A description of all covariates tested
☐	☒	A description of any assumptions or corrections, such as tests of normality and adjustment for multiple comparisons
☐	☒	A full description of the statistical parameters including central tendency (e.g. means) or other basic estimates (e.g. regression coefficient) AND variation (e.g. standard deviation) or associated estimates of uncertainty (e.g. confidence intervals)
☐	☒	For null hypothesis testing, the test statistic (e.g. *F*, *t*, *r*) with confidence intervals, effect sizes, degrees of freedom and *P* value noted<br>*Give P values as exact values whenever suitable.*
☒	☐	For Bayesian analysis, information on the choice of priors and Markov chain Monte Carlo settings
☒	☐	For hierarchical and complex designs, identification of the appropriate level for tests and full reporting of outcomes
☒	☐	Estimates of effect sizes (e.g. Cohen's *d*, Pearson's *r*), indicating how they were calculated

Our web collection on statistics for biologists contains articles on many of the points above.

Software and code

Policy information about availability of computer code

Data collection	Detailed information were provided in the materials and method section. Briefly, BD FACS Diva and FACSuite Software was used for collecting FACS Data. Bulk RNA-sequencing was performed using a HiSeq2500 (Illumina) or a NextSeq 500 (Illumina) platform. Single-cell RNA-sequencing was performed using a Novaseq6000 (Illumina).
Data analysis	For Statistical analysis, GraphPad Prism 10.0.2 was used.<br>For FACS analysis, FlowJo (v10) was used.<br>For a cytometric bead array, FCAP Array software (BD Biosciences) was used.<br>For bulk RNA-seq, TopHat (version 2.1.1) was used to map to the reference genome (UCSC/hg19) with annotation data from iGenomes (Illumina). Gene expression levels were quantified using Cuffdiff (Cufflinks version 2.2.1). Other tools used: FastQC v0.11.8, SAMtools v1.9, BEDtools v2.27.1.<br>For functional profiling: g:Profiler web server;<br>For Gene Set Enrichment Analysis (GSEA): GSEA 4.3.2 software;<br>For single-cell RNA-sequencing and analysis: Cell Ranger pipeline v.3.0.2a; R version 4.1.1.; Seurat 4.0.5.

For manuscripts utilizing custom algorithms or software that are central to the research but not yet described in published literature, software must be made available to editors and reviewers. We strongly encourage code deposition in a community repository (e.g. GitHub). See the Nature Portfolio guidelines for submitting code & software for further information.

Data

Policy information about availability of data

All manuscripts must include a data availability statement. This statement should provide the following information, where applicable:

- Accession codes, unique identifiers, or web links for publicly available datasets
- A description of any restrictions on data availability
- For clinical datasets or third party data, please ensure that the statement adheres to our policy

The raw sequence data have been deposited in the DNA Data Bank of Japan (DDBJ) database under the association number PRJDB15883 [https://ddbj.nig.ac.jp/resource/bioproject/ PRJDB15883], composing of the following subseries: DRA016355, DRA016356, DRA016432. The project is also available at the NCBI BioProject [http://www.ncbi.nlm.nih.gov/bioproject/] under the same association number. The processed data sets were deposited with the association codes E-GEAD-615 [https://ddbj.nig.ac.jp/public/ddbj_database/gea/experiment/E-GEAD-000/E-GEAD-615], E-GEAD-616, E-GEAD-617. Source data are provided with this paper. The authors declare that all data supporting the findings of this study are available within the article and its Supplementary Information file.

Research involving human participants, their data, or biological material

Policy information about studies with human participants or human data. See also policy information about sex, gender (identity/presentation), and sexual orientation and race, ethnicity and racism.

Reporting on sex and gender	n/a
Reporting on race, ethnicity, or other socially relevant groupings	n/a
Population characteristics	n/a
Recruitment	n/a
Ethics oversight	The ethics committees of Kyoto University (Kyoto, Japan) and Chiba University (Chiba, Japan) approved this study.

Note that full information on the approval of the study protocol must also be provided in the manuscript.

Field-specific reporting

Please select the one below that is the best fit for your research. If you are not sure, read the appropriate sections before making your selection.

☒ Life sciences ☐ Behavioural & social sciences ☐ Ecological, evolutionary & environmental sciences

For a reference copy of the document with all sections, see nature.com/documents/nr-reporting-summary-flat.pdf

Life sciences study design

All studies must disclose on these points even when the disclosure is negative.

Sample size	No statistical methods were used to predetermine sample size for experiments. The sample sizes were based on those generally employed in the field. All the data include at least three biological replicates.
Data exclusions	All the data were included in the analysis.
Replication	All experiments, with the exception of single-cell RNA-sequencing, were conducted in a minimum of three biological replicates. All findings were successfully replicated or reproduced at least three times in independent experiments. The single-cell RNA-sequencing analysis was performed once due to costs.
Randomization	All the samples were randomly allocated into experimental groups. There is no bias when performing each experiment and collecting data.
Blinding	The investigators were not blinded since analyses relied on unbiased measurements of quantitative parameters. However, standardized procedures for data collection and analysis were used to prevent bias.

Reporting for specific materials, systems and methods

We require information from authors about some types of materials, experimental systems and methods used in many studies. Here, indicate whether each material, system or method listed is relevant to your study. If you are not sure if a list item applies to your research, read the appropriate section before selecting a response.

Materials & experimental systems

n/a	Involved in the study
☐	☒ Antibodies
☐	☒ Eukaryotic cell lines
☒	☐ Palaeontology and archaeology
☒	☐ Animals and other organisms
☒	☐ Clinical data
☒	☐ Dual use research of concern
☒	☐ Plants

Methods

n/a	Involved in the study
☒	☐ ChIP-seq
☐	☒ Flow cytometry
☒	☐ MRI-based neuroimaging

Antibodies

Antibodies used	APC anti-human CD34 (Biolegend, Cat#343510, clone 581), APC anti-human CD41 (Biolegend, Cat#303710, clone HIP8), PE anti-human CD42b (Biolegend, Cat#303906, clone HIP1), BV421 mouse anti-human CD62P (BD Biosciences, Cat#564038), FITC-PAC-1 (BD Biosciences, Cat#340507), Anti-RalB antibody(Merck Millipore, Cat#04-03, clone 25, dilution 1:3000), and goat anti-mouse secondary antibody, Alexa Fluor™ 647(Invitrogen, Cat# A-21235,dilution 1:1000).
Validation	Antibodies were validated by manufacturers or validated in previous studies. Statements on antibody validation are present on the manufacturer's websites along with relevant references. Additional validation was done by the use of negative control (control IgG) for FACS.

Eukaryotic cell lines

Policy information about cell lines and Sex and Gender in Research

Cell line source(s)	Human ESC line KhES-3
Authentication	The human ESC line KhES-3 was obtained from the Institute for Frontier Medical Sciences, Kyoto University (Kyoto, Japan). The use of cells was approved by the ethics committees at Kyoto University and Chiba University.
Mycoplasma contamination	Mycoplasma is regularly tested and the cell lines used in this study were negative for mycoplasma contamination.
Commonly misidentified lines (See ICLAC register)	The cell line used in this study is not present in the registry of commonly misidentified lines

Flow Cytometry

Plots

Confirm that:

☒ The axis labels state the marker and fluorochrome used (e.g. CD4-FITC).

☒ The axis scales are clearly visible. Include numbers along axes only for bottom left plot of group (a 'group' is an analysis of identical markers).

☒ All plots are contour plots with outliers or pseudocolor plots.

☐ A numerical value for number of cells or percentage (with statistics) is provided.

Methodology

Sample preparation	For flow cytometry analysis, cultured and transfected cells were washed once with PBS. Then, the cells were suspended in staining medium. When required, the cells were incubated for 30 min with appropriate antibodies on ice, and the cells were filtered through a mesh filter before flow cytometry analysis. The iPSC derived platelets were incubated for 30 min with appropriate antibodies at room temperature before flow cytometry analysis.
Instrument	BD FACS Aria II, BD FACS Aria IIIu, BD FACS Verse
Software	FACS Diva or FACSuite application were used for collecting FACS Data. Data was then analyzed using FlowJo software v10
Cell population abundance	Cell sorting efficiency was confirmed by flow cytometric analysis of post-sorted cells.
Gating strategy	The cells were first gated based on FSC-A/SSC-A. Then, the cells were gated based on FSC-A/FSC-H to exclude the doublets . The cell populations in the singlet gate were used for downstream analysis. Living cells were gated based on propidium iodide (PI) negative.

☒ Tick this box to confirm that a figure exemplifying the gating strategy is provided in the Supplementary Information.
