# Supplementary material for: A let-7 microRNA-RALB axis links the immune properties of iPSC-derived megakaryocytes with platelet producibility
Source: Nat Commun. 2024 Mar 22;15:2588. doi: 10.1038/s41467-024-46605-0 (PMC10960040; doi:10.1038/s41467-024-46605-0)
Supplement: Supplementary file 2 — Peer Review File [file 41467_2024_46605_MOESM2_ESM.pdf]

nature portfolio

Peer Review File

A let-7 microRNA-RALB axis links the immune properties of iPSC-derived megakaryocytes with platelet producibility

Open Access This file is licensed under a Creative Commons Attribution 4.0 International License, which permits use, sharing, adaptation, distribution and reproduction in any medium or format, as long as you give appropriate credit to the original author(s) and the source, provide a link to the Creative Commons license, and indicate if changes were made. In the cases where the authors are anonymous, such as is the case for the reports of anonymous peer reviewers, author attribution should be to 'Anonymous Referee' followed by a clear attribution to the source work. The images or other third party material in this file are included in the article's Creative Commons license, unless indicated otherwise in a credit line to the material. If material is not included in the article's Creative Commons license and your intended use is not permitted by statutory regulation or exceeds the permitted use, you will need to obtain permission directly from the copyright holder. To view a copy of this license, visit http://creativecommons.org/licenses/by/4.0/.

Editorial Note: Parts of this Peer Review File have been redacted as indicated to maintain the confidentiality of unpublished data.

REVIEWER COMMENTS

Reviewer #1 (Remarks to the Author):

This is an interesting study demonstrating that microRNA control of expression of the small GTPase RalB leads to phenotypic decisions in megakaryocytes allowing for selection of platelet production and immune functions. The work is novel and uses an interesting miRNA approach to reveal the major candidate gene, RALB, in this phenotypic switching. The work builds upon the substantial advances that the senior author, Dr Eto, has made in generating human platelets from the human iPSC line that his team have generated, iMMKCLs, and is a further important step in refining the generation of large numbers of human platelets in vitro.

Comments to be addressed

1. The abstract needs more clearly to state whether increased or decreased RALB expression leads to subpopulation differentiation. I find the abstract unclear in this regard.
2. Does expression of RALB regulate, in some way, expression of p53 and/or CDKN1A? This is important to know, since the authors in the introduction state that certain imMKCL clones displaying cellular senescence exhibit a diminished capacity to generate iPSC-PLTs, but that this capacity can be restored through the knockdown of p53 and CDKN1A.
3. Functionally (from Fig. 6), can you antagonise the effect of the specific miRNA by overexpression of RALB?
4. Also from Fig. 6, can the authors show protein-level data for RALB expression, as well as mRNA. This will be important to do, to be sure that the transcript changes make appropriate changes in protein expression, and to definitely tie the miRNA data and function to RalB.
5. There are now inhibitors of Ral GTPases, which would be important to characterize in this system. Does the RAL inibitor Rbc8 then also regulate MK phenotype?
6. What are the effects of altered RalB expression on the platelet generated in this system? There is published data from Ral KO mouse platelets that there is a marked effect on P selectin dynamics, specifically. Stimulated plasma membrane expression of P selectin is markedly diminished in the mouse Ral KO. Are there changes in P selectin response in PLTs generated from these iMMKCLs, or from the iMMKCLs themselves?
7. From the data shown in Fig. 7, is IL8 expression causative for the phenotypic shift in immkcls overexpressing ralb? Can you force phenotype shift by culturing with IL8? And can an IL8 inhibitor block the phenotype shift induced by RALB overexpression? In addition, does IL8 explain the effects of the miRNA also?

Reviewer #2 (Remarks to the Author):

Overall an interesting study with clear data and conclusions. The data is sound, the conclusions are appropriate, and the methodology is clear and rigorous. I have only minimal comments that may help to

increase the impact and more broad translation of the study on the field.

1. Are the platelets made from the different types of Mks based on let-7 expression different? Do the platelets from cluster 3 for example have more PF4? Or similar gene expression to possible function correlations?

2. Similarly, Mk functions are implied from the RNA-seq, but can some of it be validated?

Reviewer #3 (Remarks to the Author):

This manuscript studies the role microRNAs in megakaryocyte development. The authors utilize their previously published stem cell derived megakaryocyte line, imMKCLs. Using a microRNA screening procedure, they find that let-7 influences megakaryocyte development, influencing the formation of the immune skewed megakaryocyte. Follow up experiments show RALB as a potential let-7 target, leading to the impact of let-7 on the formation of immune skewed megakaryocytes. Overall, while the studies presented are very interesting, there is overlap between let-7 effects on inflammatory megakaryocytes and megakaryocyte maturation and its unclear how this mechanism functions in primary megakaryocytes. See below for specific comments.

1) The authors show that let-7 inhibition, figure4, leads to upregulation of both inflammatory genes and megakaryocyte maturation genes. This is somewhat confusing as the good clones (high let-7) tend to demonstrate good proliferation but also good maturation. How does let-7 inhibition impact proliferation and platelet yield?

Also, what is the impact of let-7 overexpression? Can poor clones with low let-7 (7-3) be rescued by let-7 overexpression?

2) The authors argue that let-7 targets RALB, leading to its downregulation which then in turn limits the generation of inflammatory megakaryocytes. RALB was found to be upregulated in group 3 from their sc-RNA seq dataset. This group was also enriched in genes related to megakaryocyte maturation. Is RALB only working on the inflammatory targets, but also regulating maturation genes (PF4 etc…). These genes should be examined in the RALB O/E experiments described in figure 7. Showing that RALB only influences inflammatory genes but does not impact MK maturation would be an important finding that may suggest different let-7 targets are responsible for inflammatory genes vs maturation.

Also, RALB KO/KD studies should be performed to show enhanced proliferation, better platelet generation and lower IL-8 secretion when this gene is downregulated.

3) Let-7 has a well described interaction with LIN28. How is LIN28 impacted with let-7 inhibition? Some

of the results here are somewhat surprising as LIN28 expression is associated with proliferation while let-7 expression which targets LIN28 is associated with more mature progenitors with potentially more limited proliferative capacity.

Response Letter

We would like to express our sincere gratitude for the efforts invested by the reviewers in our manuscript (**NCOMMS-23-30188**). We appreciate the constructive feedback provided, which has greatly contributed to improvement of the manuscript. We have carefully considered each point raised, conducted additional experiments, and have made corresponding revisions. The point-to-point feedback is provided as followed.

Response to specific comments

Comments are in italic blue and numbered. Our responses are in black, and quoted texts from the manuscript are underlined with revised parts in red.

Reviewer #1 (Remarks to the Author):

This is an interesting study demonstrating that microRNA control of expression of the small GTPase RalB leads to phenotypic decisions in megakaryocytes allowing for selection of platelet production and immune functions. The work is novel and uses an interesting miRNA approach to reveal the major candidate gene, RALB, in this phenotypic switching. The work builds upon the substantial advances that the senior author, Dr Eto, has made in generating human platelets from the human iPSC line that his team have generated, iMMKCLs, and is a further important step in refining the generation of large numbers of human platelets in vitro.

Comments to be addressed

1. The abstract needs more clearly to state whether increased or decreased RALB expression leads to subpopulation differentiation. I find the abstract unclear in this regard.

Thank you for your suggestion. We have modified our abstract as follows.

We recently achieved the first-in-human transfusion of induced pluripotent stem cell-derived platelets (iPSC-PLTs) as an alternative to standard transfusions, which are dependent on donors and therefore variable in supply. However, heterogeneity characterized by thrombopoiesis-biased or immune-biased megakaryocytes (MKs) continues to pose a bottleneck against the standardization of iPSC-PLT manufacturing. To address this problem, here we employed microRNA (miRNA) switch biotechnology to distinguish subpopulations of imMKCLs, the MK cell lines producing iPSC-PLTs. Upon miRNA switch-based screening, we found imMKCLs with lower let-7 activity exhibited an immune-skewed transcriptional signature. Notably, the low activity of let-7a-5p resulted in the upregulation of RAS like proto-oncogene B (*RALB*) expression, which was crucial for the lineage determination of immune-biased imMKCL

subpopulations and led to the activation of interferon-dependent signaling. The dysregulation of immune properties/subpopulations, along with the secretion of inflammatory cytokines, contributes to a decline in the quality of the whole imMKCL population.

2. Does expression of RALB regulate, in some way, expression of p53 and/or CDKN1A? This is important to know, since the authors in the introduction state that certain imMKCL clones displaying cellular senescence exhibit a diminished capacity to generate iPSC-PLTs, but that this capacity can be restored through the knockdown of p53 and CDKN1A.

We examined the bulk RNA-sequencing datasets to address the regulatory role of RALB expression on p53 and CDKN1A expression. Our findings revealed that the overexpression of RALB specifically induces TP53 expression at the proliferation stage (DOX-ON) of imMKCLs but has no significant impact on CDKN1A expression (Figure A (i) for reviewers/ editor). Moreover, we found that a TP53-related signaling pathway was induced in RALB-overexpressing imMKCLs at the maturation stage (Figure A (ii) for reviewers/editor). These results consistently support our previous report (Sone, Nakamura et al., Stem Cell Reports, 2021) that showed manipulating p53 is a key determinant in restoring the proliferation rate of imMKCLs, leading to better quality. The impact of RALB on TP53 signaling indicates the regulatory dynamics involved.

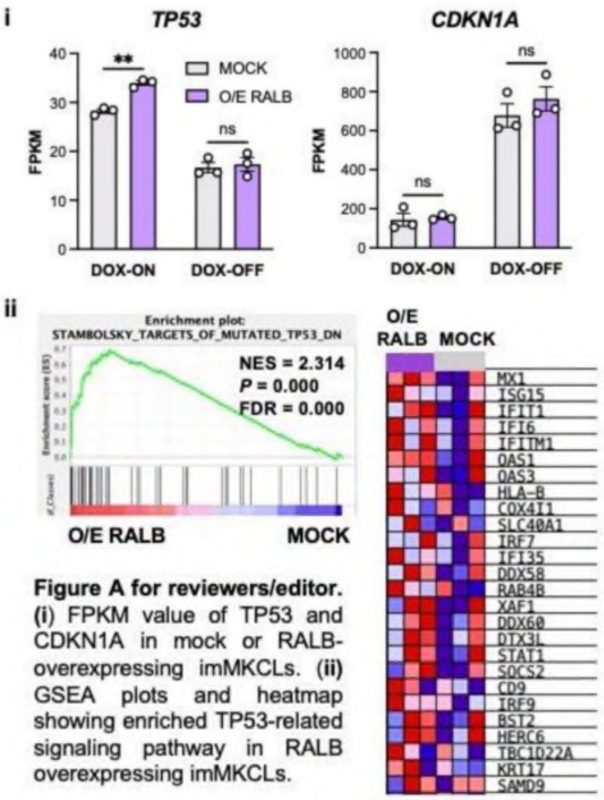

Figure A for reviewers/editor. **(i)** FPKM value of TP53 and CDKN1A in mock or RALB-overexpressing imMKCLs. **(ii)** GSEA plots and heatmap showing enriched TP53-related signaling pathway in RALB overexpressing imMKCLs.

3. Functionally (from Fig. 6), can you antagonise the effect of the specific miRNA by overexpression of RALB?

While we observed no substantial difference in the internal activity of let-7a-5p between MOCK and RALB-overexpressing cells (Figure B (i) for reviewers/editor), when we selectively sorted cells with high let-7a-5 activity and subjected them to lentiviral transfection, we found that RALB overexpression could induce immune signaling in imMKCLs (Figure B (ii-iii) for reviewers/editor). This finding indicates that the overexpression of RALB likely antagonize the effect of let-7 miRNA.

4. Also from Fig. 6, can the authors show protein-level data for RALB expression, as well as mRNA. This will be important to do, to be sure that the transcript changes make appropriate

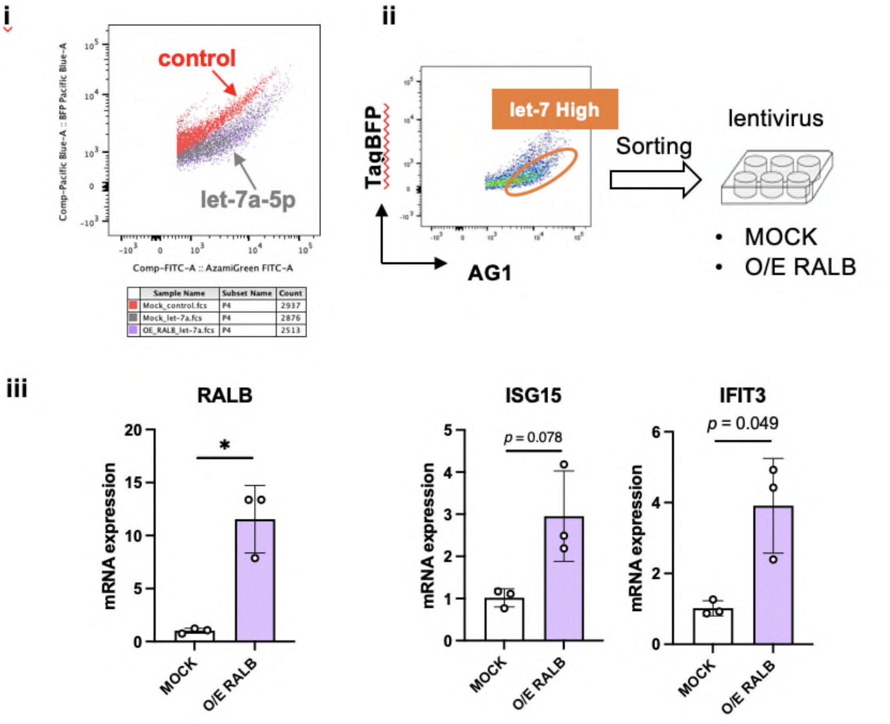

Figure B for reviewers/editor. (i) A flow cytometry analysis of let-7a-5p activity patterns in MOCK (grey) and RALB-overexpressing imMKCLs (purple). **(ii)** A schematic illustration of the sampling procedure. **(iii)** The mRNA expression of the indicated genes was increased with RALB overexpression in the let-7-high population at the proliferation stage.

changes in protein expression, and to definitely tie the miRNA data and function to RalB.

As suggested, we conducted intracellular flow cytometry and revealed elevated RALB expression at protein level in RALB-overexpressing imMKCLs compared to MOCK (new Supplementary

Fig. 4a-b). Detailed methods are provided in the revised supplementary information.

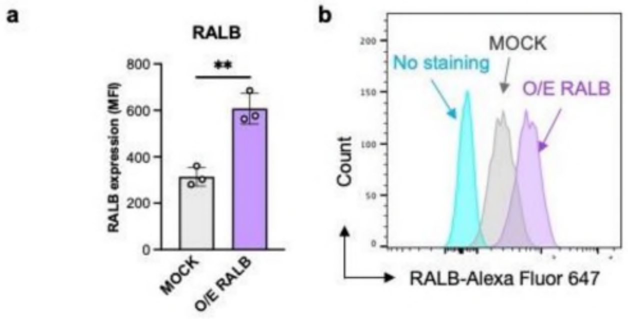

Supplementary Fig. 4
(a) The intracellular protein expression of RALB (mean fluorescence intensity, MFI) detected in MOCK or O/E RALB imMKCLs by intracellular flow cytometry. Detailed information was provided in the supplementary methods. **(b)** The RALB expression was compared using a histogram overlay.

5. There are now inhibitors of Ral GTPases, which would be important to characterize in this system. Does the RAL inhibitor Rbc8 then also regulate MK phenotype?

We purchased RBC8 (Selleckchem, S7606) and conducted additional experiments that revealed the addition of 0.1 to 1 µM RBC8 impacts the proliferation and iPSC-PLT production of imMKCLs (new supplementary figure 10). The manuscript was modified accordingly to present these findings.

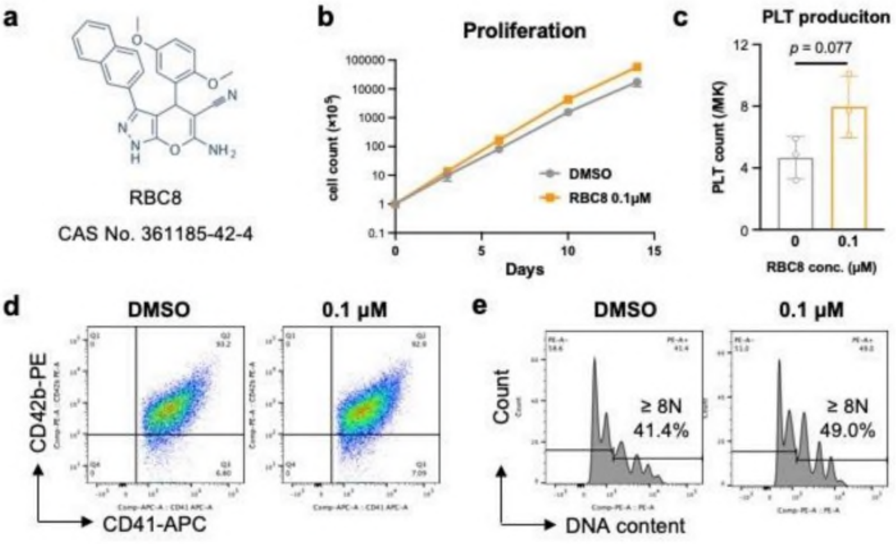

Supplementary Fig. 10
(a) Molecular structure of RBC8, a specific inhibitor of RALA and RALB. **(b)** The administration of 0.1 µM RBC8 inhibited the proliferation of imMKCLs (clone 7). (c) iPSC-PLT production under the indicated conditions. **(d)** Representative flow cytometry plots of iPSC-PLTs generated from RALB-overexpressing imMKCLs (clone 7) in the presence or absence of RBC8. **(e)** Flow cytometric analysis of ploidy.

6. What are the effects of altered RalB expression on the platelet generated in this system? There is published data from Ral KO mouse platelets that there is a marked effect on P selectin dynamics, specifically. Stimulated plasma membrane expression of P selectin is markedly diminished in the mouse Ral KO. Are there changes in P selectin response in PLTs generated from these imMKCLs, or from the imMKCLs themselves?

We show the *P*-selectin expression on iPSC-PLTs in the new supplementary figure 11. Initially, our hypothesis posited that *RALB*-overexpressing imMKCLs (low-quality) would manifest elevated *P*-selectin expression compared to MOCK imMKCLs. Unexpectedly, the iPSC-PLTs derived from both *RALB*-overexpressing imMKCLs and MOCK iMKCLs exhibited comparable *P*-selectin levels (Supplementary Fig. 11f). Notably, a low-quality clone (clone 7-3) demonstrated lower *P*-selectin expression than a high-quality clone (clone 7; Supplementary Fig. 11b). Considering this observation, we assume there might be a compensatory influence of RALB on *P*-selectin may contribute to the consistent *P*-selectin expression pattern between iPSC-PLTs generated from MOCK and O/E RALB imMKCLs.

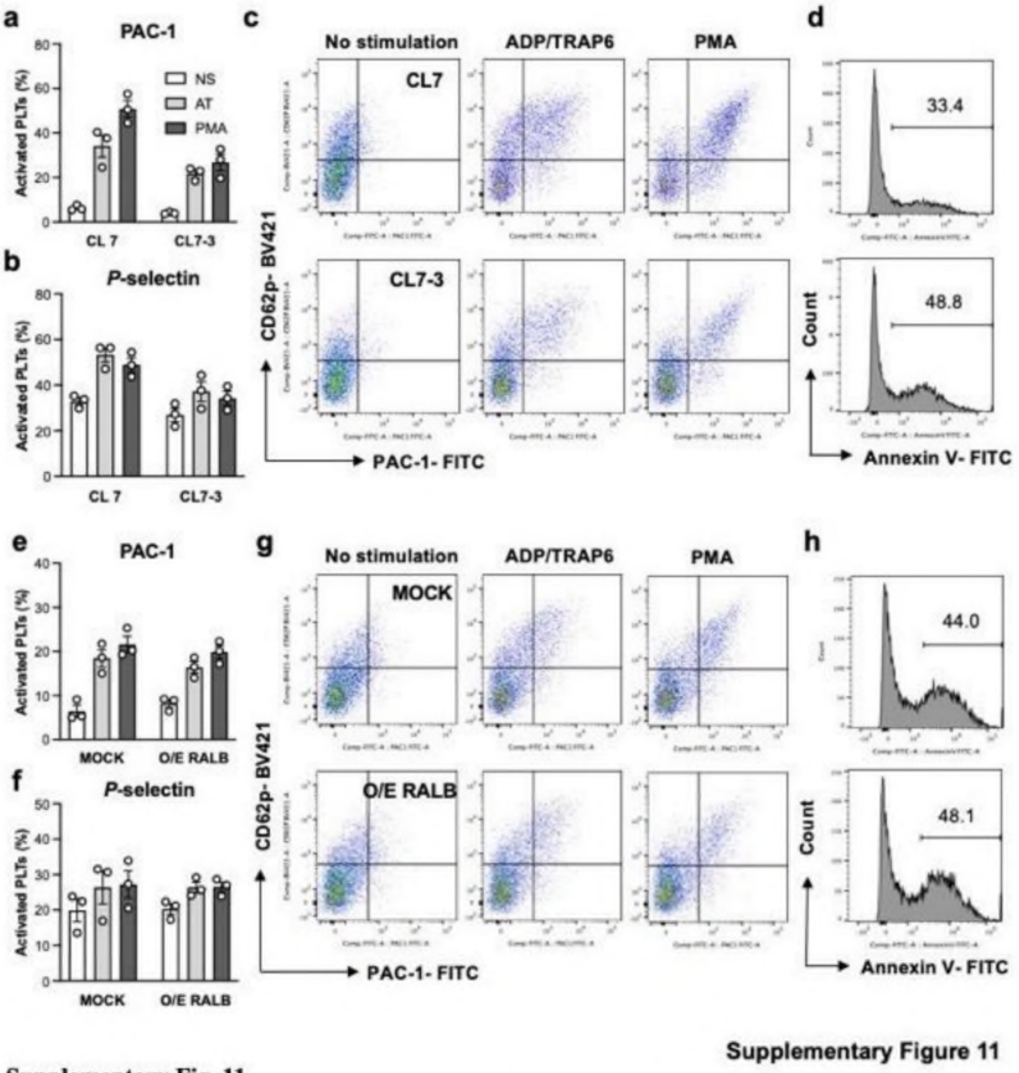

Supplementary Fig. 11
(a) PAC-1 binding and **(b)** *P*-selectin expression on iPSC-PLTs generated from clone 7 or clone 7-3 with or without PMA or ADP/TRAP6 stimulation. **(c)** Representative flow cytometry plots of activated iPSC-PLTs. **(d)** Representative histograms of Annexin V bound to CD41+ iPSC-PLTs derived from clone 7 or clone 7-3. **(e)** PAC-1 binding and **(f)** *P*-selectin expression on iPSC-PLTs generated from MOCK or RALB-overexpressing imMKCLs with or without PMA or ADP/TRAP6 stimulation. **(g)** Representative flow cytometry plots of activated iPSC-PLTs. **(d)** Representative histograms of Annexin V bound to CD41+ iPSC-PLTs derived from MOCK or O/E RALB.

7. From the data shown in original Fig. 7, is IL-8 expression causative for the phenotypic shift in imMKCLs overexpressing RALB? Can you force phenotype shift by culturing with IL-8? And can an IL-8 inhibitor block the phenotype shift induced by RALB overexpression? In addition, does IL-8 explain the effects of the miRNA also?

We purchased recombinant human IL-8/CXCL8 protein (R&D Systems) to conduct additional

experiments. We found that the addition of IL-8 into the culture diminished iPSC-PLT production (new Supplementary Fig. 7a-c) but had no significant impact on the proliferation of imMKCLs. On the other hand, blocking IL-8 signaling with Reparixin (Selleckchem, S8640), a specific CXCR1/2 inhibitor, improved iPSC-PLT production (new Supplementary Fig. 7d-g) without affecting proliferation (data not shown). A new supplementary figure 7 was provided to present these important insights.

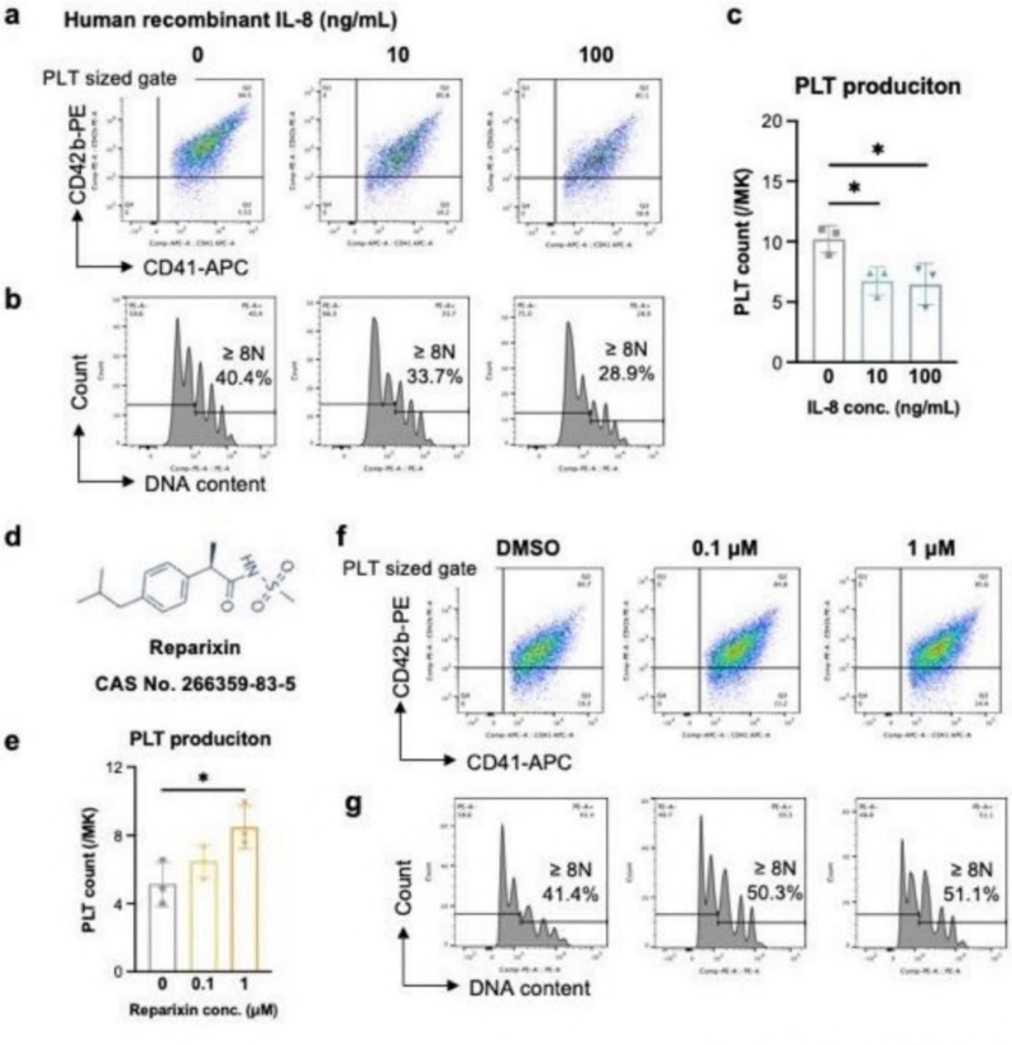

Supplementary Figure 7

Supplementary Fig. 7

(a) Representative flow cytometry plots of iPSC-PLTs generated from clone 7 in the presence of human recombinant IL-8 at the indicated concentrations. **(b)** Flow cytometric analysis of ploidy. **(c)** iPSC-PLT production under the indicated conditions. **(d)** Molecular structure of Reparixin, a specific inhibitor of CXCR1/2. **(e)** iPSC-PLT production under the indicated conditions. **(f)** Representative flow cytometry plots of PLTs generated from RALB-overexpressing imMKCLs (clone 7) in the presence of Reparixin at the indicated concentrations. **(g)** Flow cytometric analysis of ploidy. Data are expressed as the mean ± SD from three independent experiments. One-way ANOVA with multiple comparisons was used to assess statistical significance. * $P < 0.05$.

Of note, despite having distinct optimal concentrations, treatment with RBC8 or Reparxin demonstrated the capacity to restore iPSC-PLT productivity of the M35-1 clone, which is the clone utilized in the iPLAT1 clinical trial (Figure C for reviewers/editor). The consistent efficacy of these small molecules across different clones suggests their universal functionality and underscores their potential applicability in future clinical settings.

[*REDACTED*]

Reviewer #2 (Remarks to the Author):

Overall an interesting study with clear data and conclusions. The data is sound, the conclusions are appropriate, and the methodology is clear and rigorous. I have only minimal comments that may help to increase the impact and more broad translation of the study on the field.

Thank you very much for your effort and positive feedback on our revised manuscript.

1. Are the platelets made from the different types of Mks based on let-7 expression different? Do the platelets from cluster 3 for example have more PF4? Or similar gene expression to possible function correlations?

2. Similarly, Mk functions are implied from the RNA-seq, but can some of it be validated?

We incorporated the functional assay results of the generated iPSC-PLTs into new supplementary figure 11. Notably, iPSC-PLTs generated from the high-quality clone (clone 7) exhibited increased PAC-1 binding, elevated *P*-selectin expression, and reduced Annexin V binding compared to those derived from the low-quality clone (clone 7-3). However, while clones 7 and 7-3 displayed distinct let-7 activity, we currently lack direct evidence demonstrating a clear correlation between let-7 activity in imMKCLs and the functional properties of the generated iPSC-PLTs. Nevertheless, our scRNA-seq analysis identified several markers for each cluster, potentially enabling the separation of MKs with diverse function. This information may serve as a foundation for future investigations into the functional consequence of having different types of imMKCL systems as well as naïve megakaryocytes.

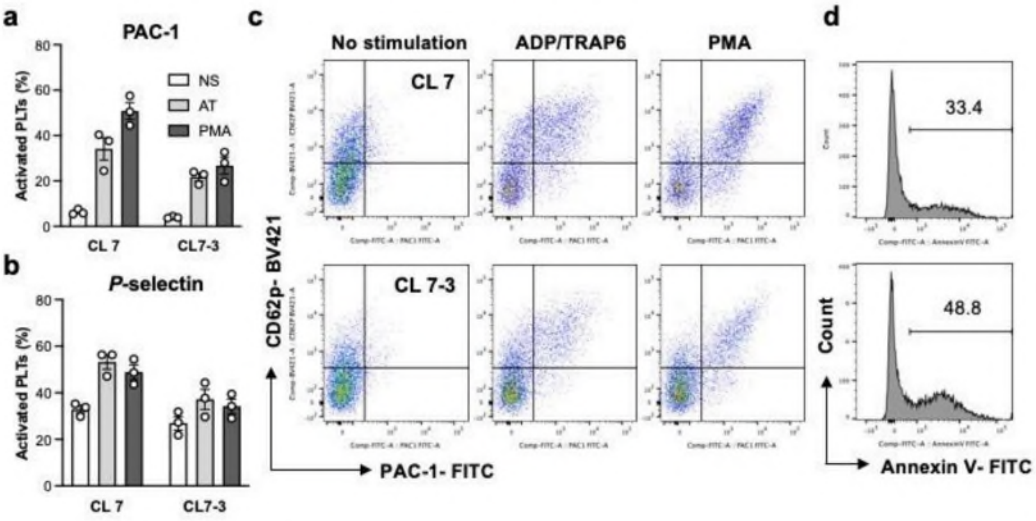

Supplementary Fig. 11 **(a)** PAC-1 binding and **(b)** *P*-selectin expression on iPSC-PLTs generated from clone 7 or clone 7-3 without or with PMA or ADP/TRAP6 stimulation. **(c)** Representative flow cytometry plots of activated iPSC-PLTs. **(d)** Representative histograms of Annexin V bound to CD41+ iPSC-PLTs derived from clone 7 or clone 7-3.

Reviewer #3 (Remarks to the Author):

This manuscript studies the role microRNAs in megakaryocyte development. The authors utilize their previously published stem cell derived megakaryocyte line, imMKCLs. Using a microRNA screening procedure, they find that let-7 influences megakaryocyte development, influencing the formation of the immune skewed megakaryocyte. Follow up experiments show RALB as a potential let-7 target, leading to the impact of let-7 on the formation of immune skewed megakaryocytes. Overall, while the studies presented are very interesting, there is overlap between let-7 effects on inflammatory megakaryocytes and megakaryocyte maturation and its unclear how this mechanism functions in primary megakaryocytes. See below for specific comments.

Thank you very much for your effort and constructive feedback on our manuscript. We employed *in vitro* differentiated megakaryocytic cells derived from cord blood-derived $CD34^+$ cells as the primary MK model to validate the observed outcomes. Although we did not observe the activation of interferon signaling upon let-7 inhibition, our investigations revealed that the overexpression of RALB upregulated interferon signaling, leading to impaired platelet generation. This finding highlighted the critical role of RALB in immune properties/subsets in MK development. New supplementary figure 11 reflects these observations.

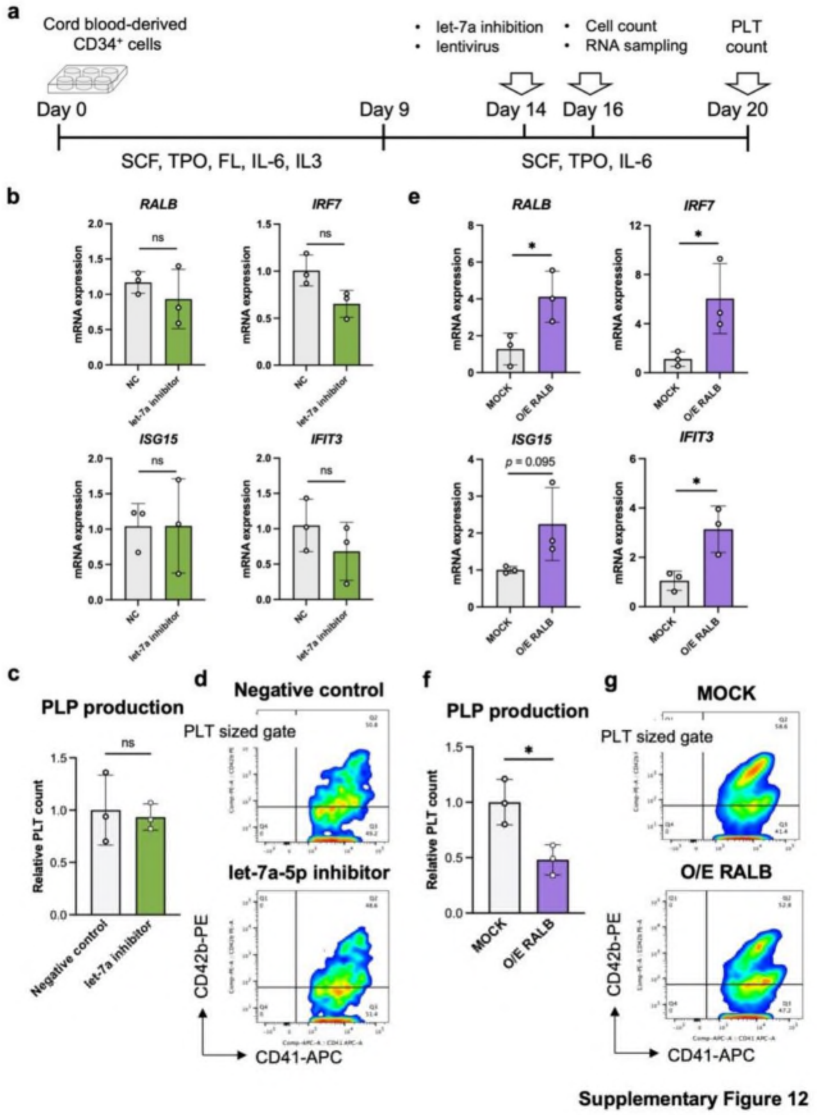

Supplementary Fig. 12

(a) A schematic illustration of *in vitro* differentiation of MKs from cord blood derived CD34+ cells. The inhibition of let-7a-5p did not have significant impact on neither mRNA expression of the indicated genes **(b)**, nor platelet-like particle (PLP) production **(c-d)**. **(e)** Significantly elevated expression levels of *RALB*, *IRF7, ISG15*, and *IFIT3* were observed in RALB-overexpressing (O/E) cells. Total RNAs were isolated from day-16 cells. The mRNA expression levels were measured by RT-qPCR and normalized to *GAPDH*. **(f-g)** The overexpression of RALB also induced a significant decline of CD41a+CD42b+ PLPs generated from cord blood-derived MKs at day 20. Data are expressed as the mean ± SEM from three independent experiments. Two-tailed student's *t*-tests were used to assess statistical significance. * $P < 0.05$.

1) The authors show that let-7 inhibition, figure 4, leads to upregulation of both inflammatory genes and megakaryocyte maturation genes. This is somewhat confusing as the good clones (high let-7) tend to demonstrate good proliferation but also good maturation. How does let-7 inhibition

impact proliferation and platelet yield?

Despite its impact on gene expression, let-7 inhibition did not cause a significant difference in the proliferation and platelet productivity of imMKCLs (Figure D (i) for reviewers/editor). This could be attributed to the intricate interplay of let-7 in regulating both the immune subset (cluster 5) and thrombopoietic subset (cluster 3). We speculate that the inhibition of let-7 may compensate for any negative impact on iPSC-PLT production induced from the immune subset by influencing the thrombopoietic subset. The observation that internal let-7 activity did not affect iPSC-PLT production (Supplementary Fig. 1c) supports this hypothesis. Accordingly, we revised the manuscript to discuss the effect of let-7 inhibition in the Result and Discussion section (page 10~12).

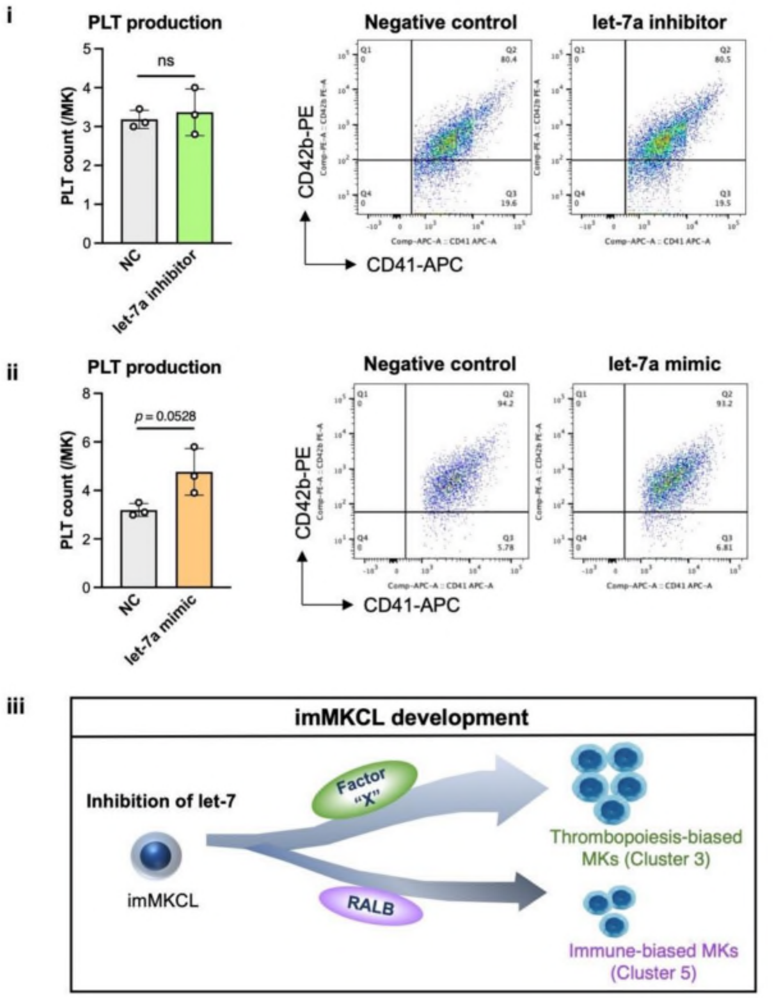

Figure D for reviewers/editor. (i) Let-7a-5p inhibition did not significantly impact the platelet production of imMKCLs. **(ii)** The overexpression of let-7a-5p by let-7 mimics slightly improved the platelet production of imMKCLs. **(iii)** Hypothesis of the regulation of let-7 miRNA in imMKCLs. Distinct let-7 downstream factors are responsible for the regulation of clusters 3 and 5.

Also, what is the impact of let-7 overexpression? Can poor clones with low let-7 (7-3) be rescued by let-7 overexpression?

We noted a modest improvement in iPSC-PLT production following let-7 overexpression, although statistical significance was not achieved (Figure D (ii) for reviewers/editor). This experiment was conducted using clone 7; the iPSC-PLT productivity of clone 7-3 cannot be rescued by let-7 overexpression, which we attribute to potential damage induced during the transfection procedure. Notably, upon transfection, clone 7-3 exhibited even lower iPSC-PLT production, making it challenging to obtain a difference in the iPSC-PLT productivity of the low-quality clone 7-3.

2) The authors argue that let-7 targets RALB, leading to its downregulation which then in turn limits the generation of inflammatory megakaryocytes. RALB was found to be upregulated in group 3 from their sc-RNA seq dataset. This group was also enriched in genes related to megakaryocyte maturation. Is RALB only working on the inflammatory targets, but also regulating maturation genes (PF4 etc.). These genes should be examined in the RALB O/E experiments described in figure 7. Showing that RALB only influences inflammatory genes but does not impact MK maturation would be an important finding that may suggest different let-7 targets are responsible for inflammatory genes vs maturation.

Thank you for the constructive comment. We carefully examined our bulk RNA-seq datasets of RALB-overexpressing imMKCLs (new supplementary figure 4) and found that the overexpression did not have a significant impact on the expression of genes associated with megakaryocyte maturation. This finding suggested that distinct let-7 targets are responsible for regulating inflammatory genes versus megakaryocyte maturation-related genes. Further, this result is consistent with our hypothesis. According to the scRNA-seq analysis, while RALB expression levels were upregulated in both clusters 3 and 5, the induced RALB expression is a decisive factor for the upregulated immune-related genes but not for the elevated MK maturation signaling. Additionally, we speculate that let-7 targets an unknown factor (Factor “X”) beyond RALB to regulate imMKCL maturation (Figure D (iii) for reviewers/editor).

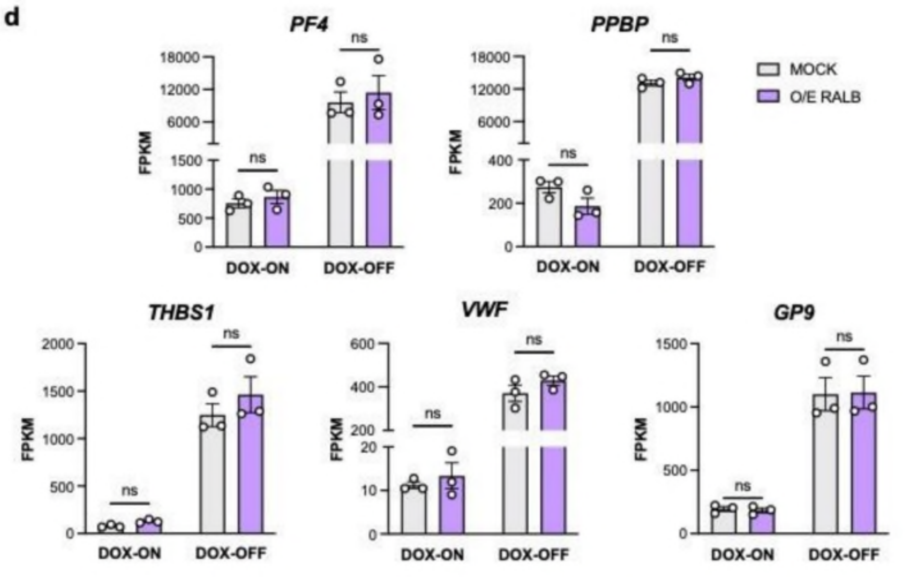

Supplementary Figure 4

Supplementary Fig.4 (d) The overexpression of RALB did not significantly impact the expression of genes related to MK maturation. The bar graphs show FPKM values of the indicated genes. Data are expressed as the mean ± SEM from three independent experiments.

Also, RALB KO/KD studies should be performed to show enhanced proliferation, better platelet generation and lower IL-8 secretion when this gene is downregulated.

Since Ral GTPases are known to function in MKs/platelets, we investigated the impact of *RALB* through knockdown experiments rather than knockout studies. To achieve *RALB* knockdown, we utilized Dharmacon siGENOME SMART pool siRNA targeting human *RALB* (Horizon Discovery). *RALB* knockdown improved the platelet production of imMKCLs while reducing the expression of interferon signaling genes (new supplementary figure 10). We revised the manuscript accordingly.

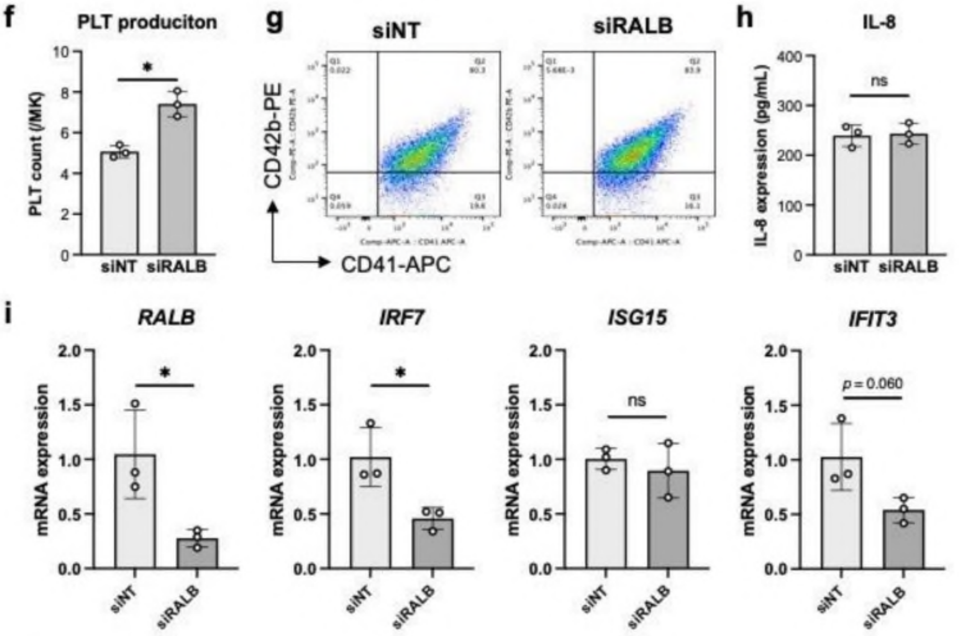

Supplementary Fig. 10

(f) siRNA-mediated *RALB* knockdown ameliorated the iPSC-PLT production from imMKCLs (clone 7). Cells were transfected with siRNA targeting *RALB* (siRALB) or non-targeting control (siNT). **(g)** Representative flow cytometry plots of PLTs generated from siNT or siRALB. **(h)** RALB knockdown did not significantly impact IL-8 secretion at the maturation stage. **(i)** The relative mRNA expression of the indicated genes was assessed in imMKCLs 48 hours following the reverse transfection procedure. mRNA expression levels were measured by RT-qPCR and normalized to *GAPDH*. Data are expressed as the mean ± SEM from three independent experiments. Two-tailed student *t*-tests were used to assess statistical significance. * $P < 0.05$.

3) Let-7 has a well described interaction with LIN28. How is LIN28 impacted with let-7 inhibition? Some of the results here are somewhat surprising as LIN28 expression is associated with proliferation while let-7 expression which targets LIN28 is associated with more mature progenitors with potentially more limited proliferative capacity.

LIN28, including LIN28A and LIN28B, is recognized as a well-established upstream regulator of the let-7 family. We found that the low let-7a-5p-activity population (let-7 low) consistently exhibited elevated LIN28A expression, which might be mediated by decreased DNA methylation in the promoter region of the gene (Figure E for reviewers/editor). This result suggests that in the context of imMKCLs, let-7a-5p miRNA activity is dependent on LIN28A levels, which themselves appear to depend on DNA methylation.

However, we did not observe a direct relationship between LIN28A with immune phenotypes of imMKCLs (Figure E (v) for reviewers/editor). We excluded this dataset from the current manuscript to ensure a focused and clear presentation.

[*REDACTED*]

REVIEWERS' COMMENTS

Reviewer #1 (Remarks to the Author):

The authors have done a lot of work to address all the comments in the review. I believe that they have fully addressed them, by changes to the manuscript but importantly in new data that are now presented, principally in the expanded Supplementary Data with some addressed to the reviewers. Figure B (for reviewers) iii is particularly important, I feel, showing that overexpression of RalB leads to a trend towards increase in immune-type expression in iMMKCLs, indicated by upregulation of expression of ISG15 and IFIT3.
Supplementary Fig. 4 addresses the comment about demonstrating upregulation of protein expression of RalB. Supplementary Fig. 10 is also important, and shows a trend to increase in platelet generation from iMMKCL cells in the presence of the Ral inhibitor RBC8, although no increase in expression of platelet markers CD41 or CD42b, or in ploidy of the cells.
Lastly, the authors have elegantly addressed the point about whether IL8 is the mediator of effects of RalB expression, and show in Supplementary Fig. 7 that an IL-8 receptor inhibitor (Reparixin) improves platelet generation, by shifting MKs away from immune-type phenotype. This is an important step forward.
Alastair Poole

Reviewer #2 (Remarks to the Author):

The authors have done a great job addressing all my concerns.

Reviewer #3 (Remarks to the Author):

My concerns have been addressed
